# Phylogeny, Histology and Inferred Body Size Evolution in a New Rhabdodontid Dinosaur from the Late Cretaceous of Hungary

**DOI:** 10.1371/journal.pone.0044318

**Published:** 2012-09-21

**Authors:** Attila Ősi, Edina Prondvai, Richard Butler, David B. Weishampel

**Affiliations:** 1 Hungarian Academy of Sciences–Eötvös Loránd University, Lendület Dinosaur Research Group, Budapest, Hungary; 2 GeoBio-Center, Ludwig-Maximilians-Universität München, Munich, Germany; 3 Center for Functional Anatomy and Evolution, Johns Hopkins University, Baltimore, Maryland, United States of America; Monash University, Australia

## Abstract

**Background:**

Rhabdodontid ornithopod dinosaurs are characteristic elements of Late Cretaceous European vertebrate faunas and were previously collected from lower Campanian to Maastrichtian continental deposits. Phylogenetic analyses have placed rhabdodontids among basal ornithopods as the sister taxon to the clade consisting of *Tenontosaurus*, *Dryosaurus*, *Camptosaurus*, and *Iguanodon*. Recent studies considered *Zalmoxes*, the best known representative of the clade, to be significantly smaller than closely related ornithopods such as *Tenontosaurus*, *Camptosaurus*, or *Rhabdodon*, and concluded that it was probably an island dwarf that inhabited the Maastrichtian Haţeg Island.

**Methodology/Principal Findings:**

Rhabdodontid remains from the Santonian of western Hungary provide evidence for a new, small-bodied form, which we assign to *Mochlodon vorosi* n. sp. The new species is most similar to the early Campanian *M. suessi* from Austria, and the close affinities of the two species is further supported by the results of a global phylogenetic analysis of ornithischian dinosaurs. Bone histological studies of representatives of all rhabdodontids indicate a similar adult body length of 1.6–1.8 m in the Hungarian and Austrian species, 2.4–2.5 m in the subadults of both *Zalmoxes robustus* and *Z*. *shqiperorum* and a much larger, 5–6 m adult body length in *Rhabdodon*. Phylogenetic mapping of femoral lengths onto the results of the phylogenetic analysis suggests a femoral length of around 340 mm as the ancestral state for Rhabdodontidae, close to the adult femoral lengths known for *Zalmoxes* (320–333 mm).

**Conclusions/Significance:**

Our analysis of body size evolution does not support the hypothesis of autapomorhic nanism for *Zalmoxes*. However, *Rhabdodon* is reconstructed as having undergone autapomorphic giantism and the reconstructed small femoral length (245 mm) of *Mochlodon* is consistent with a reduction in size relative to the ancestral rhabdodontid condition. Our results imply a pre-Santonian divergence between western and eastern rhabdodontid lineages within the western Tethyan archipelago.

## Introduction

**Figure 1:**
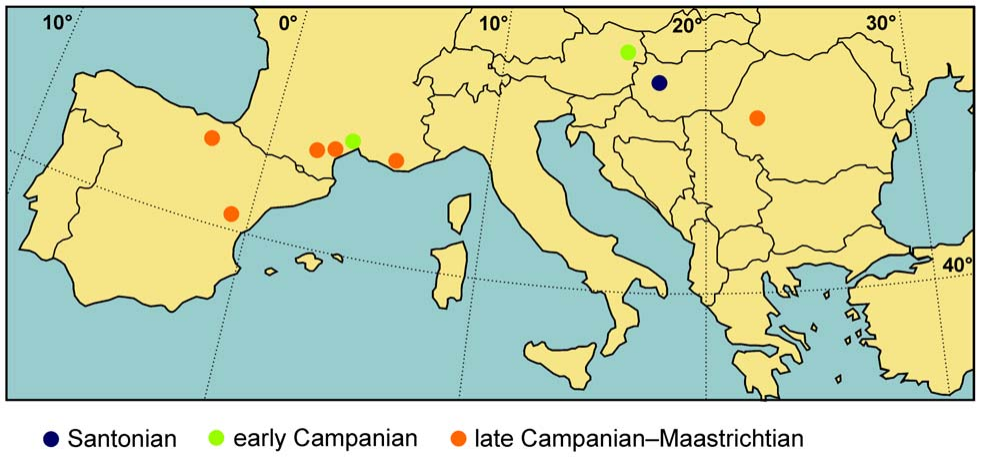
Main localities of rhabdodontid dinosaur remains in Europe. (Note that there are additional late Campanian to Maastrichtian localities in southern France).

Rhabdodontidae is a group of ornithopod dinosaurs endemic to the Late Cretaceous of Europe that has previously been considered to include two valid genera, each containing two species, known from several geographic regions ([1], Figure 1). *Rhabdodon priscus*, the first member of the group to be discovered, was unearthed close to Marseille, southern France, in the late 1840s [2], and was described by Matheron [3]. Subsequently, additional material housed in a private collection (the Panescorse Collection) was described and referred to *Rhabdodon* [4], with some additional material also being referred to this taxon by Lapparent [5]. From the 1980s onward, intensive research on various Late Cretaceous vertebrate sites in southern France resulted in a large number of new discoveries, including associated remains of *Rhabdodon* [6]–[11]. Based on a single dentary, Buffetaut and Le Loeuff [6] described *R. septimanicus*, considering it to probably represent a more robust species within *Rhabdodon*, although Allain and Pereda-Suberbiola [12] regarded it as a junior synonym of *R. priscus*. In addition to the French discoveries, specimens referred to *Rhabdodon* sp. have also been recovered from several Late Cretaceous localities in Spain (e.g. Laño, Chera), demonstrating the occurrence of the genus on the Iberian peninsula [13], [14].

A single tooth was discovered by Prof. Ferdinand Stoliczka in 1859 from the Gosau Beds (Grünbach Formation) of Campanian age, in a coal-mining district close to Muthmannsdorf, in eastern Austria. Extensive prospecting in the area by the mining administrator Pawlowitsch resulted in a large collection of bones and teeth that was first described by Bunzel [15]. In addition to the remains of various other vertebrate groups, this material contained some bones and teeth belonging to an ornithopod dinosaur. Based on their perceived close similarities with *Iguanodon*, Bunzel named the east Austrian ornithopod *Iguanodon suessii*. Seeley [16] published a revision of the specimens of Bunzel, as well as descriptions of additional material discovered from Muthmannsdorf in the 1870s. Seeley demonstrated substantial differences between *Iguanodon* and the Austrian ornithopod specimens and assigned the Austrian material to a new genus, *Mochlodon*, as the new combination *Mochlodon suessii*. Interestingly, Seeley [16] did not compare the Austrian material with the material of *Rhabdodon* described by Matheron [3]. The Austrian material was redescribed by Sachs and Hornung [17].

The next discovery of rhabdodontid remains in Europe resulted from the highly influential work of Franz Baron Nopcsa in the Haţeg Basin, Romania [18]–[22]. Originally, Nopcsa [18], [19] referred some of the non-hadrosaurian ornithopod remains from the Haţeg Basin to *Mochlodon suessi* (at that time also known from Austria) and the remaining elements to a newly erected species, *Mochlodon robustum* (amended to *M. robustus* by Weishampel et al. [1]). Later, Nopcsa suggested that the anatomical differences between *Rhabdodon* and the Transylvanian *Mochlodon* simply reflect sexual dimorphism, and referred the two Transylvanian taxa to *Rhabdodon*, as the species *R. suessi* and *R. priscum* [22]. Recent work on the Haţeg rhabdodontids indicated that their remains differ from those of *Rhabdodon* and the Austrian material (*Mochlodon suessi*); thus, Weishampel et al. [1] erected a new genus name, *Zalmoxes*, for the Haţeg rhabdodontids, and distinguished two different species: *Z. robustus* and *Z. shqiperorum*. The validity of the latter species was later supported by additional, more complete remains [23].

Here, we describe newly discovered rhabdodontid remains from the Iharkút continental vertebrate-bearing site of western Hungary [24], [25]. These remains are of Santonian age and thus represent the oldest known rhabdodontid specimens. The specimens allow a more detailed understanding of the origin and interrelationships of this endemic family of ornithopod dinosaurs. Furthermore, we present the results of an analysis of the bone histology of specimens from all known genera within Rhabdodontidae. These results not only reveal the ontogenetic stage and inferred adult body size of sampled specimens, but also the evolution of body size within the clade. This analysis allows a reassessment of the hypothesis that the Romanian rhabdodontids, *Zalmoxes* spp., represent island dwarfs [1], [26], [27].

### Institutional abbreviations

**IPB**, Steinmann Institut für Geologie, Mineralogy und Paläontologie, Universität Bonn, Germany; **MC**, Mechin Collection (private collection), Vitrolles, France; **MHN**, Muséum d'Histoire Naturelle d'Aix-en-Provence, Aix-en-Provence, France; **MTM**, Hungarian Natural History Museum, Budapest, Hungary; **NHMUK**, Natural History Museum, London, United Kingdom; **PIUW**, Paläontologisches Institut, University of Vienna, Vienna, Austria; **UBB**, Universitatea din Babes-Bolyai, Cluj-Napoca, Romania.

## Materials and Methods

### Material

Here we declare that no specific permits were required for the described field studies.

The new rhabdodontid material described here was collected during fieldwork conducted between 2001 and 2011 at the Iharkút locality, Bakony Mountains, western Hungary. All the remains collected at Iharkút are housed in the Hungarian Natural History Museum (MTM). All elements were recovered as isolated specimens from a sedimentary breccia layer that represents the richest bone-yielding horizon within the fluvial Csehbánya Formation (for geological details see [28], [29] of Santonian age [30]. Specimens were prepared mechanically in the technical labs of the Department of Paleontology of Eötvös Loránd University and the Hungarian Natural History Museum. The bones are well preserved, rich in pyrite and organic material, and black in color. The known material of this taxon exhibits varying degrees of weathering. The Hungarian rhabdodontid is represented by several skull elements, including multiple dentaries, dozens of maxillary and dentary teeth, and multiple elements of the postcranial skeleton. Some of these bones do not preserve features that have been optimized by phylogenetic analysis as rhabdodontid synapomorphies ([1], this study); they are therefore referred to this lineage based upon comparative observations (general similarities to rhabdodontids and differences from other European Late Cretaceous dinosaur groups).

### Bone histology and ontogenetic stages

The following specimens were selected for histological sampling (Table 1): (1) Six long bone specimens, including a humerus, three femora, and two tibiae, all from the Csehbánya Formation (Santonian) at Iharkút, Hungary, and referred to the new rhabdodontid species described below as *Mochlodon vorosi* n. sp.; (2) a scapula, a radius, a femur and a tibia, all from the Grünbach Formation (early Campanian) at Muthmannsdorf, Austria, and assigned to the Austrian rhabdodontid, *Mochlodon suessi* (which we resurrect here as a valid species; see below); (3) four humeri and seven femora from an early Maastrichtian grey marl level at Aix-en-Provence region (Vitrolles-Couperigne), France, all of which are assigned to *Rhabdodon*, but which are unassigned at the species level.

**Table 1:** List of sampled elements of different rhabdodontid dinosaur species used in this study.

Species/genus	Specimen number	Sampled element	Element length (mm; *estimated)	Femur length (mm; *estimated)	Estimated body length (m)	Ontogenetic stage
*Mochlodon vorosi*	MTM 2012.25.1	femur	217	217	1,6	late juvenile
	MTM 2012.26.1	tibia	179*	192*	1,4	late juvenile
	MTM V 2010.126.1	femur	160*	160*	1,2	subadult
	MTM V 01.101	tibia	148*	159*	1,2	adult
	MTM 2012.23.1	humerus	156	240*	1,8	adult
	MTM V 01.225	femur	218*	218*	1,6	adult
*Mochlodon suessi*	PIUW 3518	scapula	162*	225*	1,6	late juvenile
	PIUW 3517	radius	82*	174*	1,3	juvenile
	PIUW 2349/III	femur	105*	105*	0,8	juvenile
	PIUW 2349/35	tibia	181*	194*	1,4	adult
*Zalmoxes robustus*	FGGUB R.1392	humerus	201*	308*	2,3	late juvenile
	FGGUB R.1382	femur	280*	280*	2	subadult
	FGGUB R.1002	femur	320*	320*	2,4	subadult
*Zalmoxes shqiperorum*	FGGUB R.1088	femur	164*	164*	1,2	juvenile
	FGGUB R.1608	femur	333	333	2,5	subadult
*Zalmoxes* sp.	FGGUB R.6	humerus	180*	276*	2	subadult
	FGGUB OB 3077	humerus	255	392*	2,9	late juvenile
*Rhabdodon* sp.	MHN AIX PV 1999.12	humerus	352*	540*	4	juvenile
	MHN AIX PV 2001.12.294	humerus	236	362*	2,7	juvenile
	MHN AIX PV 2001.27	femur	513*	513*	3,7	late juvenile
	MHN AIX PV 2001.65	humerus	298	457*	3,4	juvenile
	MHN AIX PV 2001.113	femur	718*	718*	5,1	late juvenile
	MHN AIX PV 2001.A3	femur	626*	626*	4,5	juvenile
	MHN AIX PV 2007.4.115	femur	688*	688*	4,9	juvenile/late juvenile
	MHN AIX PV 2007.4.116	femur	820*	820*	5,9	adult
	MHN AIX PV 2008.1.11	femur	210	210	1,5	adult
	Mechin collection 472	humerus	326*	500*	3,7	late juvenile
	Mechin collection 676	femur	703*	703*	5	late juvenile

Samples were taken mainly from the diaphyseal regions, but consistency in sampling location was not possible due to the incompleteness, fragile nature, and/or scientific value of the specimens. To acquire entire cross sections from the fragile specimens of the Hungarian *Mochlodon vorosi* n. sp. (humerus [MTM 2012.23.1], femur [MTM 2012.25.1], tibia [MTM 2012.26.1]), the sampled regions were stabilized with resoluble resin and cut with a precision saw. In light of their diagnostic value, only small pieces from the fractured surfaces of the outer half of the cortex were extracted from two femora (MTM V 2010.126.1; MTM V 01.225), and one tibia (MTM V 01.101). Entire and half diaphyseal cross sections were made from the bones assigned to *Mochlodon suessi* without embedding them in stabilizing resin. Core samples were obtained from all *Rhabdodon* specimens following the histological core drilling method described by Stein and Sander [31]. With the exception of one longitudinal section from a broken humeral epiphysis, all samples were processed into transverse thin sections following standard methods [32]. Thin sections were studied under a Leica DMLP polarized light microscope, photographed with a Leica DFC420 digital camera, and images were obtained and processed with Imagic ImageAccess software. Interpretative figures were compiled using Photoshop CS5 and CorelDRAW X5. Published histological slides of *Zalmoxes robustus*, *Z. shqiperorum* and *Zalmoxes* sp. [26] housed at IPB were also included in the current investigation.

Based on the microstructural features of the sampled bones, a developmental state (i.e. juvenile, late juvenile, subadult or adult) was assigned to each specimen. Histological indicators used to define different ontogenetic stages are the porosity, vascular density and orientation, number and distribution pattern of LAGs, degree of secondary remodeling, and features of osteocyte lacuna discerned throughout the cortex. Neither providing growth strategy reconstructions nor performing skeletochronological analysis with absolute age estimations were among the main goals of this study. Additional information about the sampled specimens and sections is given in Table 1.

### Femur and body length estimation and reconstruction of body size evolution

To compare the body size obtained by different sampled individuals within a single corresponding ontogenetic stage, a standardised method was used to estimate the femur length for each specimen and body length for the specimens of *Mochlodon*, *Zalmoxes* and *Rhabdodon* species considered in the analysis. Rather than shaft diameter, length data were measured or estimated because most of the investigated specimens were incomplete, compressed or crushed. Complete, well-preserved elements were photographed or images were taken from the literature for rhabdodontid species, digitally measured, and line drawings prepared in different views using CorelDRAW X5. These contour-drawings of set proportions but freely adjustable dimensions were then used as reference objects to estimate the total length of homologous, but incomplete, histologically sampled skeletal elements. To provide phylogenetic context for the evaluation of body size evolution in Rhabdodontidae, published data on maximal femur lengths of phylogenetically bracketing ornithopod taxa ranging from the basal ornithopod *Orodromeus* to the ankylopollexian *Planicoxa* were collected (Table 2). Wherever possible, data were collected for specimens known to be adult on the basis of histological investigation. Total body length for each included *Mochlodon*, *Zalmoxes* and *Rhabdodon* specimens was then estimated based on skeletal reconstructions obtained from the literature (Table 2.). Estimated values of body lengths were acquired by scaling the skeletal restoration of the phylogenetically closest species to the measured or estimated size of the skeletal element concerned. This procedure was performed only once for each type of bone for each species considered. The ratio thus obtained between the length of a given skeletal element and total body length was used to calculate body length for the rest of the studied specimens of the same species. A summary and more details about data acquisition for body length estimation are provided in Table 1.

**Table 2:** List of maximum femoral lengths of different ornithopods used in this study.

Species	max femur length (mm)	Log10femur	Notes	reference	histological ontogenetic status	Formation	Age (Ma)
*Orodromeus makelai*	170	2,230448921	estimate	[68]	adult	Two Medicine Formation; middle Campanian	76
*Haya griva*	169	2,227886705		[69]	?	Javkhlant Formation; Santonian	84,5
*Changchunsaurus parvus*	157	2,195899652	estimate	[38]	?	Quantou Formation; Aptian-Albian	109
*Jeholosaurus shangyuanensis*	135	2,130333768		[39]	?	Yixian Formation; ealy Aptian	122
*Hypsilophodon foxii*	200	2,301029996		[68]	?	Wessex Formation; Barremian	127,5
*Gasparinisaura cincosaltensis*	160	2,204119983		[70]	subadult	Anacleto Formation; early Campanian	80
*Thescelosaurus neglectus*	448	2,651278014		[71]	?	Lance and Hell Creek Formations; late Maastrichtian	66
*Parksosaurus warreni*	270	2,431363764		[71]	?	Horseshoe Canyon Formation; early Maastrichtian	69
*Talenkauen santacrucensis*	500	2,698970004		[72]	?	Pari Aike Formation; early Maastrichtian	69
*Rhabdodon priscus*	600	2,77815125		[8]	?	Campanian–Maastrichtian of Spain + France	71
*Rhabdodon* sp.	820	2,913813852		current study	adult	Campanian–Maastrichtian of Spain + France	71
*Rhabdodon?*	210	2,322219295		current study	adult	Campanian–Maastrichtian of Spain + France	71
*Mochlodon suessi*	194	2,28780173		current study	adult	Grünbach Formation; early Campanian	80
*Mochlodon vorosi*	240	2,380211242		current study	adult	Csehbanya Formation; Santonian	84,5
*Zalmoxes shqiperorum*	333	2,522444234		current study	subadult	Densus Ciula Formation; late Maastrichtian	66
*Zalmoxes robustus*	320	2,505149978		[26]	subadult	Densus Ciula Formation; late Maastrichtian	66
*Tenontosaurus tilletti*	580	2,763427994		[68]	subadult	Cloverly Formation; late Aptian–middle Albian	110
*Tenontosaurus dossi*	577	2,761175813		[73]	?	Twin Mountains Formation; Aptian	118,5
*Dryosaurus altus*	490	2,69019608		[68]	subadult	Morrison Formation; Kimmeridgian–Tithonian	150,5
*Callovosaurus leedsi*	280	2,447158031		[74]	?	Oxford Clay Formation; Callovian	163
*Dysalotosaurus lettowvorbecki*	350	2,544068044		[75]	adult	Tendaguru Formation; Kimmeriddian	153
*Valdosaurus canaliculatus*	432	2,635483747		[33]	?	Wessex Formation; Barremian	127,5
*Elrhazosaurus nigeriensis*	162	2,209515015		[76]	?	Elrhaz Formation; Aptian	115
*Camptosaurus dispar*	590	2,770852012		[68]	?	Morrison Formation; Kimmeridgian–Tithonian	150,5
*Uteodon aphanoecetes*	430	2,633468456		[77]	?	Morrison Formation; Kimmeridgian–Tithonian	150,5
*Cumnoria prestwichii*	420	2,62324929		[78]	?	Kimmeridge Clay; Kimmeridgian	153
*Planicoxa venenica*	520	2,716003344		[79]	?	Cedar Mountain Formation; Barremian	127,5

To test whether there is numerically-detectable evidence of autapomorphic and/or phyletic nanism within Rhabdodontidae, as reported by Benton et al. [26] (see also [27]), we reconstructed body size evolution among basal ornithopods. To do so, we expanded the results of the phylogenetic analysis within Ornithopoda by including five dryosaurid taxa (topology taken from Barrett et al. [33]; *Kangnasaurus* was excluded due to its highly uncertain stratigraphic position) and several basal ankylopollexian taxa for which body size proxies were available (*Camptosaurus dispar*, *Uteodon aphanoecetes*, *Cumnoria prestwichii*, *Planicoxa venenica*; topology taken from [34]). *Zephryosaurus* was excluded due to the lack of published postcranial material. For each of the 25 ornithopod taxa in the resulting topology we collected body size data, in the form of log_10_maximum femoral length (estimated maximal femur length based on an specifically indeterminate *Zalmoxes* humerus, FGGUB OB 3077 was excluded from the analysis), and stratigraphic range (data modified from the *Paleobiology Database*). The phylogeny was calibrated against time with taxa assigned absolute ages by taking the range midpoint. Unconstrained/zero length branches were given a length by setting a root length (arbitrarily set at 10 million years) and sharing this time equally between unconstrained branches, using the date.phylo function of Graeme Lloyd (http://www.graemetlloyd.com/methdpf.html). Mesquite 2.75 was then used to reconstruct ancestral states for femoral length using weighted squared-change parsimony. In addition, we also carried out a modified analysis in which *Rhabdodon* was split into small (maximum femoral length: 210 mm) and large species (maximum femoral length: 820 mm), based upon histological observations. Nomenclature used to describe body size evolution follows that of Gould and MacFadden [35].

### Phylogenetic analysis

To assess the phylogenetic positions of the rhabdodontid taxa discussed here we carried out two separate phylogenetic analyses, using phylogenetic datasets that contain a substantial sampling of basal ornithopods as well as basal iguanodontians. We did not utilise the recent iguanodontian phylogeny of McDonald [36] because of its currently limited sampling among non-iguanodontian ornithopod species. First, we modified the basal ornithopod matrix of Weishampel et al. [1], adding to it four new characters as well as *Mochlodon vorosi*, for a complete dataset of 79 characters and 19 taxa (see Appendix 1 for the new characters and data matrix and Appendix 2 for character matrix of Weishampel et al. [1]). The data matrix was analyzed using the heuristic search algorithm of PAUP 4.0 beta 10 for Windows [37] with default settings. All characters were treated as unordered and unweighted.

We also carried out a second analysis using the ornithischian data matrix of Butler et al. [38], as modified by Han et al. [39] (see Appendix 3 for character list). We added seven new characters (two of them were also included in the first analysis described above, these are characters 232 and 233) and split the supraspecific taxon Rhabdodontidae up into five species-level operational taxonomic units: *Rhabdodon priscus*, *Mochlodon suessi*, *Mochlodon vorosi*, *Zalmoxes robustus*, and *Zalmoxes shqiperorum*. The resultant data matrix consists of 233 characters and 58 taxa (see Appendix 4: note that an all-zero ‘dummy’ character was added at the beginning of the matrix to aid with interpretation because the computer program TNT numbers characters beginning with ‘0’). Six characters (character numbers 112, 135, 137, 138, 174, 228) were treated as ordered, as in previous iterations of this analysis [38].

The matrix was analysed using TNT [40]. First, we analyzed the matrix under the ‘new technology search’ option using sectorial search, ratchet, tree drift, and tree fuse options with default parameters and 100 random addition sequences. Second, these generated trees were analysed under traditional TBR branch swapping (which more fully explores each tree island). Standard bootstrapping (sampling with replacement) was carried out using 1,000 replicates and a new technology search (ratchet, with 10 random addition sequences). Reduced bootstrap standard frequencies were calculated excluding five wildcard taxa (see results).

### Nomenclatural Acts

The electronic version of this document does not represent a published work according to the International Code of Zoological Nomenclature (ICZN), and hence the nomenclatural acts contained in the electronic version are not available under that Code from the electronic edition. Therefore, a separate edition of this document was produced by a method that assures numerous identical and durable copies, and those copies were simultaneously obtainable (from the publication date noted on the first page of this article) for the purpose of providing a public and permanent scientific record, in accordance with Article 8.1 of the Code. The separate print-only edition is available on request from PLOS by sending a request to PLOS ONE, 1160 Battery Street, Suite 100, San Francisco, CA 94111, USA along with a check for $10 (to cover printing and postage) payable to “Public Library of Science".

In addition, this published work and the nomenclatural acts it contains have been registered in ZooBank, the proposed online registration system for the ICZN. The ZooBank LSIDs (Life Science Identifiers) can be resolved and the associated information viewed through any standard web browser by appending the LSID to the prefix “http://zoobank.org/". The LSID for this publication is: urn:lsid:zoobank.org:pub:361B072E-B46E-42F4-B28D-05F598878385.

## Results

### Systematic palaeontology

Ornithischia Seeley, 1887 [41]

Ornithopoda Marsh, 1881 [42]

Iguanodontia Sereno, 1986 [43] (sensu Sereno 2005 [44])

Rhabdodontidae Weishampel, Jianu, Csiki & Norman, 2003 [1]

*Mochlodon* Seeley, 1881 [16]

#### Type species

*Iguanodon suessii* Bunzel [15], later recombined as *Mochlodon suessii* by Seeley [16], as *Mochlodon suessi* by Nopcsa [18], as *Mochlodon suessi* by Weishampel et al. [1], and as *Rhabdodon suessi* by Steel [45] and Pincemaille-Quillévéré [9]. The type material was referred to *Zalmoxes* sp. by Sachs and Hornung [17].

#### Lectotype

Right dentary (PIUW 2349/2) [17].

#### Type locality

Konstantin mining tunnel, Felbering Mine, Muthmannsdorf, Wiener Neustadt-Land district, Niederösterreich (Lower Austria), Austria.

#### Type horizon

Grünbach Formation, Gosau Group, lower Campanian.

#### Diagnosis

Small-bodied rhabdodontid dinosaur with a total body length of approximately 1.5–2 meters distinguished from *Rhabdodon* and *Zalmoxes* on the basis of the following unique combination of characters (autapomorphies marked with an asterisk): mandibular symphysis is only slightly curved medially; *dorsal margin of the symphyseal region has a deep and caudally wider groove; *depression (depth ranging from 1–3 mm) on the lateral wall of the caudal part of the dentary, just below the coronoid process, that becomes more obvious in larger individuals; *the dorsal edge of the sympysis in lateral view is directed straight rostrally or slightly rostroventrally (in *Mochlodon suessi*), parallel to the long axis of the dentary;.

#### Remarks

Following the work of Seeley [16] and the early works of Nopcsa [18], [19], the material of *Mochlodon suessi* from Austria was referred to *Rhabdodon* by most authors [9], [20], [45], [46]. However, Weishampel et al. [1] and Weishampel and Jianu [27] regarded *Mochlodon suessi* as a nomen dubium because they considered the Austrian material to be non-diagnostic. Sachs and Hornung [17] redescribed the Austrian material and concluded that, although in their opinion indeterminate, it is more similar to the Transylvanian rhabdodontid *Zalmoxes* than to *Rhabdodon*. As a result, they referred the Austrian material to *Zalmoxes* sp. Thus, the Austrian material has been referred on at least one occasion to every genus in Rhabdodontidae during the last 135 years, and still there is no consensus concerning its taxonomic status. The Hungarian material described here helps to clarify this problem because it is not from *Rhabdodon* or *Zalmoxes*, but is most similar to the Austrian remains (see below). This similarity is further supported by the close palaeogeographic position (<100 km) of the two localities during the Late Cretaceous, and their similar stratigraphic age. Based on autapomorphic features of the dentary (not recognized by Sachs and Hornung [17]), we here resurrect the generic name *Mochlodon* for the Austrian (early Campanian) and Hungarian (Santonian) material, but distinguish two different species based upon osteological differences of the dentaries (see below).

### *Mochlodon suessi* (Bunzel 1871, [14])

#### Lectotype

Right dentary (PIUW 2349/2).

#### Type locality

As for the genus.

#### Type horizon

As for the genus.

#### Diagnosis

The dentary of *Mochlodon suessi* differs from that of the Hungarian species *Mochlodon vorosi* n. sp. (see below) in having the dorsal margin of the symphyseal region slightly rostroventrally oriented and its rostral tip in a deeper position.

#### Referred material

Dentary tooth (PIUW 2349/3); maxillary tooth (PIUW 2349/4); fragmentary parietal (PIUW 2349/54); fragmentary left scapula (PIUW 3518); fragmentary ?radius (PIUW 3517); ?manual ungual (PIUW 2349/38); fragmentary left femur (PIUW 2349/3); fragmentary ?right tibia (PIUW 2348/35) [16].

#### Remarks

The lectotype of *Mochlodon suessi* is one of the smallest rhabdodontid dentaries (74 mm preserved length) that might well represent a juvenile specimen.

### *Mochlodon vorosi* n. sp

ZooBank LSID for species.

urn:lsid:zoobank.org:act:0C76CFEA-53E7-44E2-82D8-73DE0A7C21AE

#### Holotype

Left complete dentary with four broken teeth (MTM V 2010.105.1).

#### Etymology

In honour of Dr. Attila Vörös, palaeontologist and full member of the Hungarian Academy of Sciences who founded the Paleontological Research Group of the Hungarian Academy of Sciences.

#### Type locality

Iharkút, Veszprém County, Bakony Mountains, Transdanubian Range, western Hungary.

#### Type horizon

Csehbánya Formation, Santonian [30].

#### Referred specimens

Left postorbital (MTM 2012.14.1); two right quadrates (MTM V 2010.110.1, V 2010.111.1), two left (MTM V 2010.105.1., 2012.15.1) and two right (MTM V 2010.107.1., V 2010.109.1.) dentaries, all four of which are almost complete, six fragmentary dentaries (MTM V 2010 106.1, V 2010 107.1, V 2010 108.1, V 2010 109.1, V 2010.112.1, 2012.16.1), 15 maxillary and 23 dentary teeth (MTM V 2000.01., V 2000.32., V 2000.33., V 2003.10., V 01.161., V 2003.14,–V.2003.16, V 01.64., 2012.17.1, 2012.18.1), isolated cervical (MTM 2012.19.1), dorsal (MTM 2010.118.1.), and caudal (MTM 2012.20.1, 2012.21.1) vertebrae, almost complete but compressed sacrum (MTM V 2010.121.1.), three coracoids (MTM V 01.53., V 2010.122.1., V 2010.123.1.), one fragmentary scapula (MTM 2012.22.1), one fragmentary (MTM 2012.23.1) and one complete humerus (MTM V 2010.128.1.), one complete ulna (MTM 2012.24.1), two almost complete femora (MTM V 01.225., V 2010.126.1.), one fragmentary femur (MTM 2012.25.1), one complete tibia (MTM V 2010.127.1.), two fragmentary tibiae (MTM V 01.101., 2012.26.1), and two phalanges (MTM 2012.27.1, 2012.28.1).

#### Diagnosis

*Mochlodon vorosi* n. sp. differs from *Mochlodon suessi* in having a dentary with a markedly deeper depression just below the coronoid process that becomes transversely shallower but dorsoventrally wider toward the dentary–surangular suture. The rostral tip of the dentary is directed rostrally (rather than being rostroventrally directed as in *M. suessi*), such that the dorsal margin of the symphyseal region is horizontal and thus close to the level of the alveolar margin. This difference can also be observed between the smallest dentary of *M. vorosi* and the lectotype of *M. suessi* confirming a genuine taxonomical rather than ontogenetic feature. The groove on the dorsal margin of the symphyseal region is bordered caudally by a dorsally rounded vertical wall that separates the first alveolus from the symphyseal region. *Mochlodon vorosi* can further be distinguished from species of *Rhabdodon* and *Zalmoxes* in that the proximal end of the quadrate of *M. vorosi* is strongly curved caudally (directed caudodorsally at c. 60° to the vertical plane) compared to that of *Zalmoxes robustus* (c. 45°), *Zalmoxes shqiperorum* (c. 20°) and *Rhabdodon* sp. (c. 25° in specimen MC 397).

### Description and comparisons

#### Cranial remains

**Figure 2:**
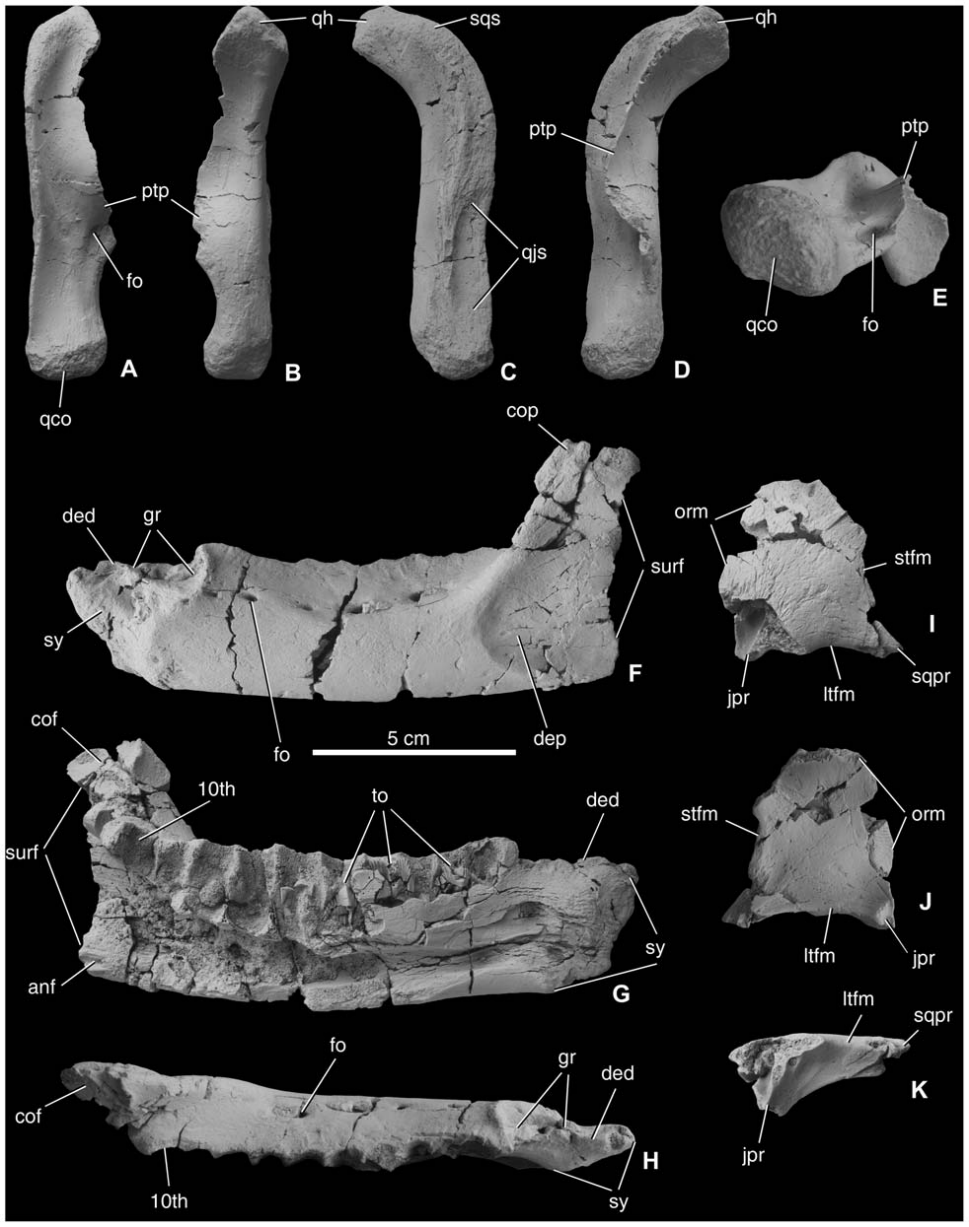
Cranial remains of *Mochlodon vorosi* n. sp. from the Upper Cretaceous Csehbánya Formation, Iharkút, western Hungary. A, right quadrate (MTM V 2010.111.1) in cranial, B, caudal, C, lateral, D, medial, E, distal views; F, left dentale (MTM V 2010.105.1) in lateral, G, medial, H, occlusal views; I, left postorbital (MTM 2012.14.1) in dorsal, J, ventral, K, lateral views. Anatomical abbreviations: anf, articular surface for angular; cof, articular surface for coronoid; cop, coronoid process; ded, dorsal edg of the dentary; dep, depression; fo, foramen; gr, groove; jpr, jugal process; ltfm, margin of lateral temporal fenestra; orm, orbital rim; ptp, pterygoid process; qco, quadrate condyles; qh, quadrate head; qjs, articular surface for quadratojugal; sqpr, squamosal process; sqs, articular surface for squamosal; stfm, margin of supratemporal fenestra; surf, articular surface for surangular; sy, symphysis; to, tooth; 10th, 10th alveolus.

*Quadrate* (Figure 2A–E). Two right quadrates of *Mochlodon vorosi* are known, with the most complete one (MTM V 2010.111.1) being slightly smaller (total length 90 mm). These quadrates show several important differences compared to that those *Zalmoxes* and *Rhabdodon*, including features that can be used as diagnostic charaters of *M. vorosi*. In general, the quadrate of *M. vorosi* (the quadrate of *M. suessi* is unknown) is more gracile than in *Zalmoxes robustus* (NHMUK R3393) and *Z. shqiperorum* (UBB NVZ1-39), and in this respect it is more similar to the unpublished specimen referred to *Rhabdodon* sp. (MC 397). In rostral and caudal views, the quadrate shaft is straight. On its rostrolateral surface is a well-developed, slightly concave articulation surface for the quadratojugal that ends just below the mid-height of the quadrate shaft. The rostral margin of this articular facet is straight and extends rostral to the quadrate condyles, unlike the condition in *Zalmoxes* (NHMUK R3393, UBB NVZ1-39) and *Rhabdodon* (MC 397). On the proximolateral surface of the bone is the contact surface for the squamosal. The head of the quadrate at the proximal end of the bone is small, slightly convex in lateral view and dorsoventrally elongated, similar to *Zalmoxes* (NHMUK R3393, UBB NVZ1-39). The distal end of the quadrate is not as wide and robust as in *Zalmoxes* but is rather small and slightly rostrally curved in lateral view, similar to *Dryosaurus altus* from the Upper Jurassic of the USA [47]. Whereas in *Rhabdodon* (MC 397), and especially in *Zalmoxes* (NHMUK R3393, UBB NVZ1-39), this distal end is asymmetrical with a distally more strongly developed lateral condyle, in *M. vorosi* the two condyles are small, are positioned at the same level in caudal view, and no intercondylar groove can be observed. In *Zalmoxes* a ridge extends along the shaft of the quadrate on its caudal surface that terminates distally at the heel of the medial mandibular condyle [1]. This ridge is not so prominent in *Rhabdodon* (MC 397) and terminates instead above the lateral condyle. In *M. vorosi*, however, this ridge is not present. Medially, the thin and plate-like pterygoid ala of the quadrate is only partially preserved in both quadrates known for *M. vorosi*. Caudally, this region is strongly concave. Ventrally the pterygoid ala is thickened, but the medially oriented process present in *Zalmoxes* [1] is not preserved in *M. vorosi*. On the rostral side of this thickened ventral region of the ala is a small, but marked, depression.

*Postorbital* (Figure 2I–K). A left postorbital is relatively completely preserved. This small (rostrocaudal length of 34 mm), thin, plate-like bone shows a marked inflexion laterally that represents the border between the dorsal and lateral surfaces of the skull. The surface of the postorbital is generally smooth, with tiny grooves present on its external surface. The postorbital is triradiate with medial, ventral and caudal processes that would have connected with the frontal, jugal and squamosal, respectively. Rostrally the orbital margin is almost straight, sharp and not as thick and rugose as in *Zalmoxes* [1], [23]. The frontal process is dorsoventrally thin and rostrocaudally wide (23 mm). The caudal process is triangular in cross section and its ventral surface bears a rostrocaudal, grooved, scarf facet for the squamosal, similar to *Zalmoxes* [1]. Laterally, the ventral process is triangular in cross section, and just above this process a channel-like opening enters the body of the postorbital medially and slightly caudomedially. The curved ledge between the jugal and squamosal processes of the postorbital in both species of *Zalmoxes* [1], [23] is also present in *Mochlodon vorosi*. This ledge forms the dorsal margin of the rostrodorsal margin of the infratemporal fenestra, and is generally smooth but ornamented with a few, very shallow ridges. A small neurovascular foramen is present just above the jugal process. This slightly concave surface may have been the origin of parts of the external adductor musculature [1]. Godefroit et al. [23] suggested it as a potential synapomorphy of *Zalmoxes*, but it might instead represent a character linking *Zalmoxes* and *Mochlodon*.

**Figure 3:**
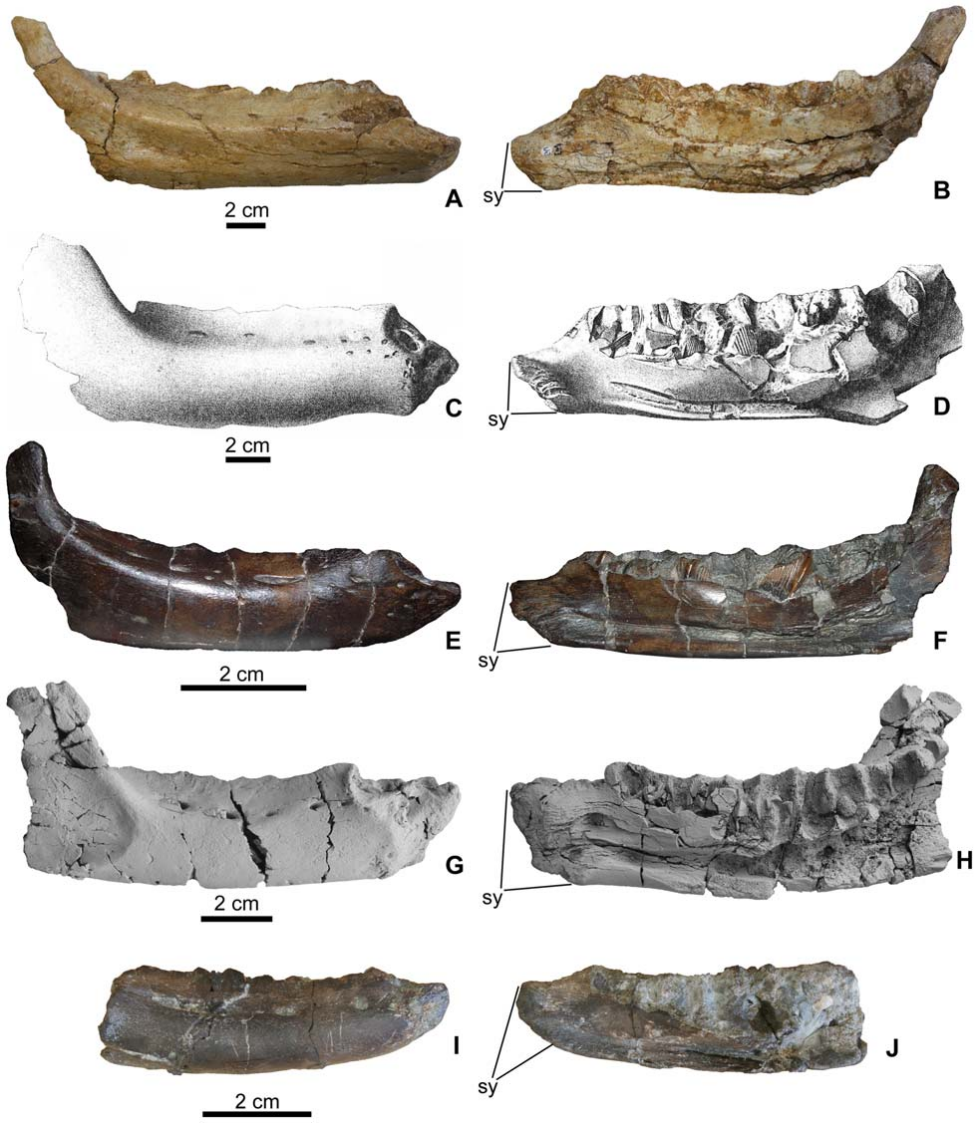
Comparison of rhabdodontid dentaries. A, *Rhabdodon* sp. (MC 443) in lateral, B, medial views; C, *Zalmoxes robustus* (NHMUK R4912) in lateral, D, medial views; E, *Mochlodon suessi* (PIUW 2349/2) in lateral, F, medial views; G, *Mochlodon vorosi* n. sp. (MTM V 2010.105.1) in lateral, H, medial views; I, Smallest dentary of *Mochlodon vorosi* n. sp. (MTM V 2010.109.1) in lateral, J, medial views. Anatomical abbreviation: sy, smyphysis.

*Dentary* (Figure 2F–H). The ten complete or partial dentaries of *Mochlodon vorosi* represent at least part of an ontogenetic series and provide insights into ontogenetic changes in its anatomy. Whereas the largest dentary (MTM V 2010.105.1) is 13.2 cm long, the estimated length of the smallest specimen (MTM V 2010.109.1) is about 65 mm (the dentary of the lectotype of *M*. *suessi* is 74 mm). All of the larger specimens contain 10 alveoli. The smallest dentary (MTM V 2010.109.1, Figure 3I, J) bears at least eight alveoli, and, although broken caudally, on the basis of the position of the last alveolus it appears that this was the last or penultimate tooth position, indicating a lower tooth count (eight or nine) in smaller individuals, similar to the ontogenetic changes observed in *Zalmoxes robustus* [48]. The general morphology and shape of the dentary of *M. vorosi* is similar to that of *Rhabdodon* and *Zalmoxes*, in that the main body of the bone is relatively straight in lateral view with parallel dorsal and ventral margins. The dorsal margin is very gently concave rostrocaudally and the ventral margin very slightly convex (Figure 3). In small individuals (MTM V 2010.109.1, Figure 3I, J), the dentary has a more strongly convex ventral margin, similar to that of *Zalmoxes* [1]. The dentary of *Zalmoxes* (especially that of *Z*. *shqiperorum*), [23] is proportionally shorter and more robust than that of *Mochlodon* spp. and *Rhabdodon priscus* (MC 443).

The symphyseal part of the dentary of *Mochlodon vorosi* bears several diagnostic features. The symphysis of *M*. *vorosi* is deeper dorsoventrally than in any of the other rhabdodontids, including *M*. *suessi*. This region is not inclined rostroventrally and slightly medially in lateral view as in *Zalmoxes* or in *Rhabdodon* but is instead directed straight rostrally and is dorsoventrally deep with its rostralmost point positioned far dorsally at the same level as the alveoli (Figure 3). As a result of this morphology, the symphyseal facet is more extensive dorsoventrally than, and not as ventrally positioned, as in *Zalmoxes* and *Rhabdodon*. In the type specimen, the rostroventral edge of the symphysis bears a small, pointed protuberance that is not as well developed in smaller individuals (e.g. MTM V 2010.109.1). The dorsal margin of the symphyseal region of *Mochlodon* is different than that of *Zalmoxes* and *Rhabdodon* (Figure 3). It bears a rostrocaudally elongate and deep groove that widens caudally. Whereas in smaller individuals of *M. vorosi* (and also in the lectotype specimen of *M*. *suessi*) this caudal region is only a few millimetres wider than the rostral part of the groove; in the largest specimens (e.g. the holotype) the groove becomes a wide (c. 10 mm) and shallow circular depression. This groove contains several neurovascular foramina that are also present in this region in *Zalmoxes robustus*, although in *Z. robustus* the foramina are not set in a groove [1], [18]. In *M. vorosi*, a large neurovascular foramen is present just ventral to this groove on its lateral side and opens rostrally.

In dorsal view, the dentary is straight with a wide buccal shelf just lateral to the alveolar margin, as occurs in other rhabdodontids. The tooth row extends nearly parallel to the lateral surface of the dentary. Depending on the size of the dentaries, the lateral surface of this buccal shelf is pierced by three (on the smallest specimen) to six (on the largest specimens) neurovascular foramina, among which the more caudal foramina are always rostrocaudally elongated and sometimes groove-like. Caudally, the buccal shelf becomes a slightly concave platform that separates the caudal three alveoli from the laterally offset coronoid process. Relative to the length of the dentary, this buccal platform is not as wide as in *Z*. *shqiperorum* [23]. The caudolateral surface of the dentary bears a depression in both species of *Mochlodon*, but it is significantly deeper in *M*. *vorosi* than in *M*. *suessi* (Figure 3E–H). The rostral margin of this depression is at the level of the eighth alveolus, and caudally, toward the dentary–surangular suture, it becomes transversely shallower and dorsoventrally wider. Fine, rostrocaudally-oriented ridges ornament the surface of this depression. The role of this depression is unclear, but it may have served as an extended insertion area for parts of the external jaw adductor musculature that usually attach on the lateral and dorsal surfaces of the coronoid eminence/region of archosaurs [49]. If this is the case, then *Mochlodon* may have possessed a highly derived external jaw adductor musculature compared to other rhabdodontids. On the dentary of *M. suessi* only the very rostral end of this depression can be observed, and it is relatively shallow. In the holotype of *M. vorosi*, all surfaces on the dentary that formed articular contacts with other bones are preserved. The dentary–surangular contact is a waved and denticulate suture with a mediolaterally wider and concave cotylus-like surface at its caudodorsal end. The contact surface for the coronoid is a flat, obliquely oriented surface on the medial side of the coronoid process. The posterodorsally oriented coronoid process of *M*. *vorosi* appears to be more similar to those of *Rhabdodon* sp. or *Z*. *robustus* (Figure 3) than to the almost vertically oriented process of *M*. *suessi* or Z. *shqiperorum*. The almost 2 cm long dentary–angular suture is positioned on the medioventral surface of the caudoventral corner of the dentary. This surface bears at least one prominent longitudinal ridge. Rostral to the rostral end of the dentary–angular articulation the medial surface of the ventral margin of the dentary forms a flat, rugose surface up to the level of the third alveolus; this surface represents the the contact for the splenial. There is no indication that the external mandibular fenestra was present in *Mochlodon*. In medial view, the rostral part of the mandibular adductor fossa is present at the caudal end of the dentary, and is continuous rostrally with the mandibular canal. This canal becomes dorsoventrally narrower and transversely shallower rostrally and terminates just caudal to the symphyseal facet.

**Figure 4:**
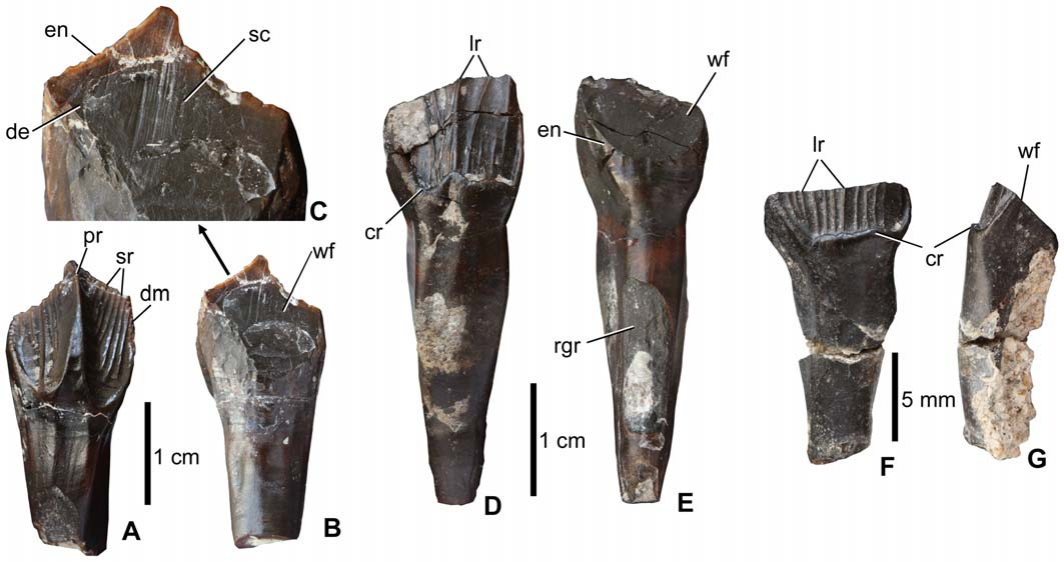
Teeth of *Mochlodon vorosi* n. sp. from the Upper Cretaceous Csehbánya Formation, Iharkút, western Hungary. A, dentary tooth (MTM 2012.18.1) in lingual, B, labial views, C, details of the labially positioned wear facet. D, maxillary tooth (MTM 2012.17.1) in labial, E, lingual views; F, strongly worn maxillary tooth (MTM 2012.17.1) in labial, G, ?mesial views. Anatomical abbreviations: cr, crenelated ridge; de, dentine, dm, denticulated margin; en, enamel; lr, longitudinal ridge; rgr, groove to accomodate the margin of crown of replacement tooth; pr, primary ridge; sc, scratch; sr, secondary ridge; wf, wear facet.

*Teeth* (Figure 4). Maxillary and dentary teeth of rhabdodontid dinosaurs are relatively common elements at Iharkút. These teeth are very similar to those of *Zalmoxes* and *Rhabdodon*, and most of them bear well-developed wear facets. Unworn maxillary tooth crowns are asymmetrical in labial or lingual view with the apex of the crown offset mesially or distally. Enamel covers the crown on all sides (Figure 4D–E), but labially it is much thicker than lingually. The labial surface is ornamented by 8–13 parallel ridges, which are more-or-less parallel to one another (MTM 2012.17.1). In unworn teeth, the ridges culminate in denticles along the mesial and distal margins of the crown, similar to the condition in *Zalmoxes* [1]. These labial ridges are generally subequal in size, but on some of the maxillary teeth one of the centrally positioned ridges is more strongly developed and raised above the other ridges, but not as strongly developed as the primary ridge of the dentary teeth (see below). The mesial and distal margins of the crown bear denticles even in those parts where no ridge terminates. Basally, the labial enamel surface is bordered by a crenelated ridge that curves apically along the mesial and distal margins. However, the crown is not transversely expanded above the root in mesial or distal view, and so the ‘cingulum’ differs from the structure that is referred to as a ‘cingulum’ in basal ornithischian dinosaurs [50]. The lingual surface is convex and ornamented by various, subparallel, faint ridges that not as strongly developed as those on the labial surface. Wear facets are positioned on the lingual surface of the crown (Figure 4E). In the early stages of wear there are frequently paired mesial and a distal facets that sometimes merge together in more heavily worn teeth. Whereas in the early stages of wear the facets form an angle of approximately 65–70° to the horizontal plane, in heavily worn teeth this angle is 35–45° (Figure 4E, G). Similarly to *Zalmoxes* [1], scratches on the worn dentine surface are vertically oriented and more-or-less parallel with one another indicating an orthal movement during dental occlusion.

As in other rhabdodontids, dentary teeth differ from the maxillary teeth in having a well developed and massive, centrally positioned, primary ridge on their lingual surfaces (MTM 2012.18.1, Figure 4A). This ridge divides the lingual surface into two slightly concave, U-shaped surfaces. Each of these surfaces bears 5–7 secondary ridges that, similar to those of the maxillary teeth, terminate along the mesial and distal edges of the tooth crown. Basally, the crowns do not possess a crenelated ridge, unlike the condition in the maxillary teeth, and the secondary ridges usually do not reach the basal margin of the U-shaped enamel surface. On the mesial and distal surfaces of the crown, a slightly denticulate margin is present. In all preserved dentary teeth, the labial surface bears a well-developed, steeply inclined wear facet that forms an angle of 10–20° to the vertical plane (Figure 4B). Similar to the maxillary teeth, two separate wear facets were formed in the early stages of wear, which became confluent in the later stages. Some teeth show marked vertically oriented scratches on the dentine that are up to 5 mm in length (Figure 4C). Whereas the root of the maxillary teeth is three times longer than the crown, that of the dentary teeth is only 1–1.5 time longer. Grooves are present on the lingual surface of the root in both maxillary and dentary teeth, and were formed by the gradual eruption of the replacement teeth.

#### Axial skeleton

**Figure 5:**
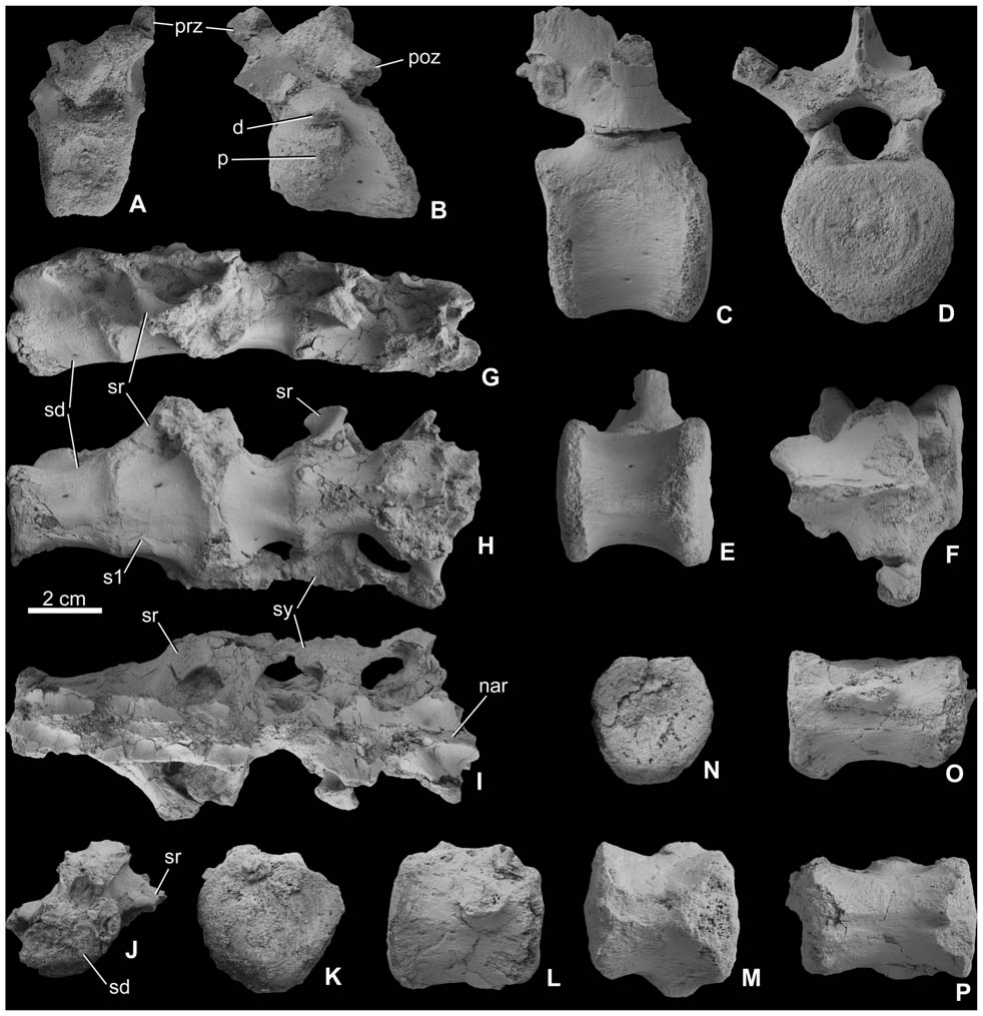
Vertebrae of *Mochlodon vorosi* n. sp. from the Upper Cretaceous Csehbánya Formation, Iharkút, western Hungary. A, cervical vertebra (MTM 2012.19.1) in cranial, B, lateral views; C, dorsal vertebra (MTM 2010.118.1.) in lateral, D, cranial, E, ventral, F, dorsal views; G, sacrum (MTM 2010.118.1.) in left lateral, H, ventral, I, dorsal, J, cranial views. K, caudal vertebral centrum (MTM 2012.20.1) in ?proximal, L, lateral, M, ventral views; N, caudal vertebral centrum (MTM 2012.21.1) in ?proximal, O, lateral, P, ventral views. Anatomical abbreviations: d, diapophysis; nar, neural arch; p, parapophysis; poz, postzygapophysis; prz, prezygapophysis sd, sacrodorsal vertebra; sr, sacral rib; sy, sacral yoke.

*Cervical vertebrae* (Figure 5A, B). A single cervical vertebra (MTM 2012.19.1) is here referred to *Mochlodon vorosi*. The neural spine and the ends of three of the zygapophyses are broken, but the vertebra is otherwise complete and well preserved. It has an amphycoelous centrum that is longer than high, with a slightly trapezoidal caudal articular surface. The cranial and caudal articular surfaces of the centrum are not parallel to one another; instead, the centrum is much longer along its ventral margin than dorsally, similar to the morphology of the fourth vertebra of *Hypsilophodon foxii* [51]. This indicates that a distinct curature was present in the cervical series of *Mochlodon vorosi*. On its ventral surface the vertebra bears a ventral keel, similar to that of *Zalmoxes* [1]. The prezygapophyses are notably longer than the postzygapophyses. The diapophyses are placed laterally on the base of the neural arch, whereas the parapophyses are short and stocky bumps placed on the dorsolateral surface of the cranial half of the centrum.

*Dorsal vertebrae* (Figure 5C–F). From the dorsal series, only a few isolated and fragmentary vertebrae are known and are mostly eroded centra. The most complete (MTM 2010.118.1.) is very similar to that of *Zalmoxes* [1], [22]. The centrum is approximately as long as high, transversely compressed at the midpoint of its axial length, and keeled along its ventral surface. This keel is not straight but slightly concave in lateral view. The articular surface of the centrum is platycoelous to slightly amphicoelous and has a circular to slightly oval outline (taller than wide). The articular surfaces are not parallel with each other in lateral view, but form an angle of approximately 5° to one another, so that the ventral margin of the centrum is somewhat shorter axially than the dorsal margin, similar to the centra figured by Nopcsa [22]. Similar to other rhabdodontids, these vertebral proportions would have resulted in an arched dorsal vertebral column. Laterally, the centrum bears two small (1 mm in diameter) neurovascular foramina on each side. The neural canal is circular and 8 mm wide. The neural arch (excluding the neural spine) is axially shorter than the centrum. The transverse processes are orientated at approximately 60° relative to the vertical plane. They are axially wider basally and become narrower and more pointed toward their distal ends. Neither the diapophyses nor the parapophyses are preserved on any dorsal vertebra referred to *M*. *vorosi*. On the most complete specimen, only the left postzygapophysis is partially preserved. In the same specimen, only the base of the neural spine (app. 1.3 cm high) is preserved, so that the complete dorsal extension of the neural spine is unknown.

*Sacrum* (Figure 5G–J). An almost complete but dorsoventrally compressed sacrum (MTM 2010.121.1.) is referred here to *Mochlodon vorosi*. As preserved, it is composed of five fused vertebrae, but caudally it is broken. As a result, the total number of vertebrae in the sacral sequence is unknown (in *Zalmoxes robustus* at least eight sacral vertebrae are present: one fused dorsal, one sacrodorsal, three true sacrals and three sacrocaudals, [1]). The sacrum of *M. vorosi* is generally similar to that of *Zalmoxes*, but a few differences are observed. The neural spine is broken and incomplete in all of the sacral vertebrae, but at least at their bases the spines were separate from each other. The ventral or ventrolateral surfaces of all of the sacrals bear one or two small neurovascular foramina. All vertebrae are connected to one another via a thickened intervertebral suture. The sacrum is slightly arched dorsally in lateral view, but due to the postmortem deformation of the bones the original shape cannot be determined. In contrast to both species of *Zalmoxes*, the last dorsal vertebra is not fused to the sacrum [1], [23]. The first element of the preserved sacral series can be regarded as a sacrodorsal, because it has a centrum that is slightly wider caudally than cranially and because the rib of the succeeding first true sacral vertebra has migrated cranially to fuse across the articulation between the two adjacent vertebrae. Ventrally the centrum has a shallow groove. In this sacrodorsal the neural arch is still high with dorsolaterally-oriented transverse processes. However, its neural arch is completely fused to that of the next vertebra. The second vertebra is the first true sacral, and has a strongly widened and flattened centrum with very broad articular surface for the third sacral vertebra. The sacral rib of the third vertebra is laterally directed and is fused to this massive, widened region at the contact between the second and third sacral vertebrae. The third vertebra becomes transversely narrower caudally, and a shallow groove is present on the ventral surface of the articulation with the fourth sacral vertebra. The fourth sacral vertebra is similar to the first sacral (the sacrodorsal) in having a relatively narrow centrum with a shallow groove ventrally. A short and wide laterally-oriented sacral rib is present on the craniolateral surface of the fourth sacral and is triangular in cross section. As occurs in *Zalmoxes* [1], [23], the sacral ribs of the second to fifth vertebrae expand laterally to form a sacrocostal yoke, which would have attached to the internal surface of the ilium. The fifth sacral vertebra is damaged so that its exact morphology cannot be determined, but it appears to be more expanded transversely in ventral view than the fourth sacral vertebra. A short and laterally-oriented sacral rib is present on the central part of its lateral surface. The neural arch has been strongly compressed postmortem in all sacral vertebrae, thus few details of its anatomy can be determined.

*Caudal vertebrae* (Figure 5K–P). A few isolated caudal vertebrae are tentatively referred to *Mochlodon vorosi*. Unfortunately, in most cases only the vertebral centrum is preserved, and it is not easy to identify their position within the caudal series. One of the elements (MTM 2012.20.1) is apparently from the proximal part of the caudal sequence, because it has a centrum that is only slightly longer axially than wide transversely. The proximal and distal articular surfaces are not rounded or hexagonal but are instead broadly heart-shaped and platycoelous, similar to proximal caudals of *Zalmoxes robustus* [1]. In ventral view, the centrum is slightly spool-shaped. The ventral keel is not as prominent as in the dorsal vertebrae, and it bears a slight midline furrow between the haemapophyseal facets. The proximal stumps of the incompletely preserved fused transverse processes can be observed on the dorsolateral surfaces of the centrum.

#### Appendicular skeleton

**Figure 6:**
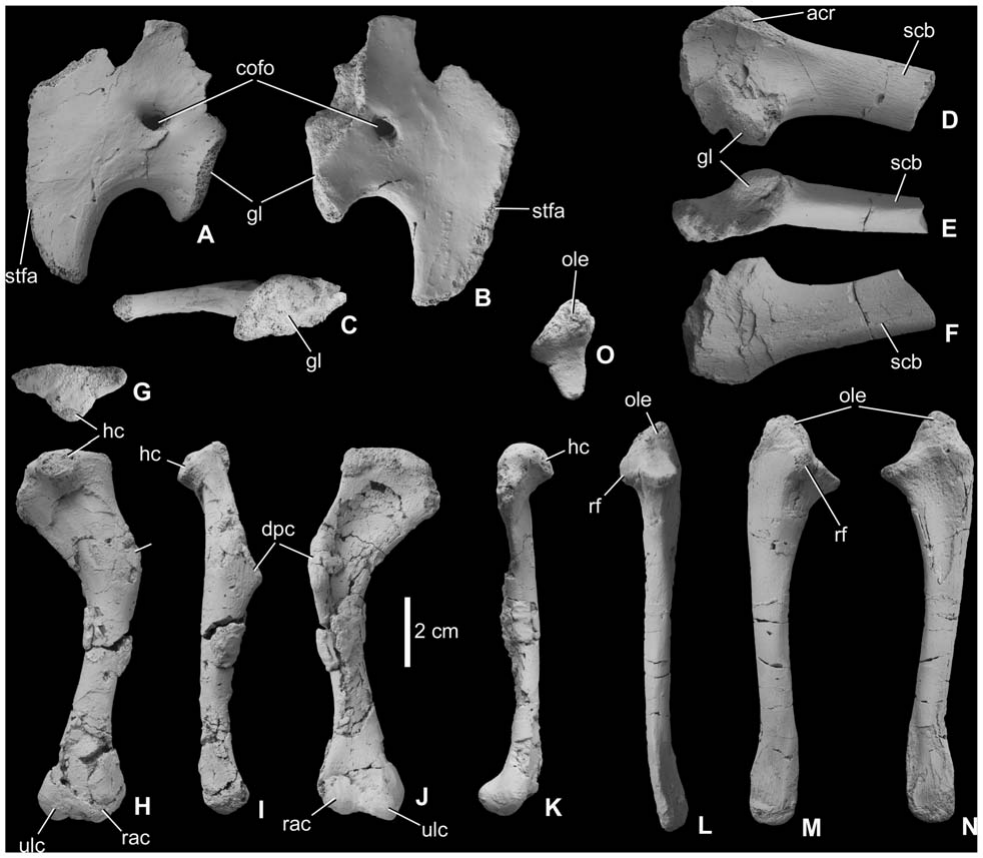
Pectoral girdle and forelimb elements of *Mochlodon vorosi* n. sp. from the Upper Cretaceous Csehbánya Formation, Iharkút, western Hungary. A, left coracoid (MTM V 01.53.) in lateral, B, medial, C, caudal views; D, left scapula (MTM 2012.22.1) in lateral, E, ventral, F, medial views; G, right humerus (MTM V 2010.128.1.) in proximal, H, caudal, I, lateral, J, cranial, K, medial views; L, right ulna (MTM 2012.24.1) in cranial, M, lateral, N, medial, O, proximal views. Anatomical abbreviations: acr, acromion process; cofo, coracoid foramen; dpc, deltopectoral crest; gl, glenoid; hc, humeral condyle; ole, olecranon process; rac, radial condyle; rf, facet for radius; scb, scapular blade; stfa, articular surface for sternum; ulc, ulnar condyle.

*Pectoral girdle (* *Figure 6A–F* *).* One incomplete left scapula (MTM 2012.22.1, Figure 6A–C) and three incomplete left coracoids (MTM V 01.53., V 2010.122.1., V 2010.123.1.) are referred to *Mochlodon vorosi*. The narrow scapula is almost identical to that of *Zalmoxes shqiperorum* (NHMUK R4900, [1], [23]) and completely differs from the relatively short, dorsoventrally wide and flattened scapula of *Rhabdodon*. In *M. vorosi*, only the proximal half of the scapular blade is preserved, and it has an oval cross section. Whereas it has a rounded dorsal margin (with the blade held horizontally) that is straight in lateral view, the ventral margin is more keeled and slightly concave in lateral view. Proximally, a shallow 2 cm long ridge is present on the dorsolateral surface of the scapular blade. Proximally, a gently concave deltoid fossa is present on the lateral surface of the scapula. This region is bordered dorsally and craniodorsally by the acromion process (Figure 6D), the dorsal edge of which is broken. Cranially, the morphology of the sutural contact with the coracoid is unclear because this margin of the bone is also broken. The scapular part of the deeply concave, oval-shaped glenoid faces ventrally.

The coracoids referred here to *Mochlodon vorosi* are very similar to those of *Zalmoxes shqiperorum*. The largest coracoid (MTM V 2010.123.1.) is slightly compressed mediolaterally. The smaller and more complete coracoid (MTM V 01.53., Figure 6A–C) is broken at its craniodorsal and dorsal margins and the articular surface for the scapula is also missing. The coracoid portion of the glenoid faces caudally and is not as concave as that of the scapula. The ventromedially-directed sternal process is straight in lateral view, and is ventrally extended and tapers to a point, forming the cranial margin of the deeply embayed coracoid notch, similarly to that of *Rhabdodon* and *Zalmoxes* [1], [23]. Whereas the coracoid body is thickened (16 mm) at the glenoid, toward its dorsal and cranial margins it becomes thinner (4–5 mm), plate-like and slightly concave on its medial surface. The subcircular coracoid foramen is placed in a more ventral position than that of *Z*. *shqiperorum* [23].

*Humerus (* *Figure 6G–K* *).* A complete right humerus (MTM V 2010.128.1., Figure 6G–J) and a fragmentary, but well-preserved left humerus (MTM 2012.23.1) are referred to *Mochlodon vorosi*. They show some differences compared to the humeri of *Rhabdodon* and *Zalmoxes*. The proximal third of the humerus is strongly bowed medially relative to the shaft of the bone (at an angle of 35–37° to the main axis of the shaft, Figure 6H, J). This curvature is approximately 10–12° in *Rhabdodon*, 8–27° in *Z*. *shqiperorum* [23] and 22–35° in *Z*. *robustus*. It is more strongly bent than in other basal ornithopods, but it is almost similar to that of *Z*. *robustus*, so this feature cannot be regarded as an autapomorphic feature of *Mochlodon*. The proximal end of the humerus of *Mochlodon* (and other rhabdodontids) is not as strongly twisted relative to the shaft as the condition in *Hypsilophodon foxii* [51]. The shaft of the bone is subcircular in cross section and much more slender relative to its total length than in other rhabdodontids. The deltopectoral crest is well developed with a straight or slightly concave lateral margin that distally has a cranial-to-cranioventrally facing, rugose surface. Laterally, this surface is separated by a longitudinally extending groove from the remainder of the shaft. The medial and lateral margins of the proximal third of the bone (the part of the bone that is strongly bent medially) diverge gently toward the proximal end. The proximal articular surface has a caudally-facing humeral head that is situated centrally on the epiphysis and is either spherical or slightly wider transversely than craniocaudally. The humeral head of *Z. shqiperorum* is spherical [23] and that of *Z. robustus* and *Rhabdodon priscus* extends farther distally along the caudal surface of the humerus than in *Mochlodon vorosi*. The distal articular surface of the humerus is formed by the well-developed ulnar and radial condyles. These condyles are separated cranially by a deep and wide intercondylar groove and ventrally by a shallow groove. Similarly to the humerus of other rhabdodontids, the ulnar condyle is more strongly developed and extends further distally than does the radial condyle.

*Ulna (* *Figure 6L–O* *).* A complete right ulna (MTM 2012.24.1) referred here to *Mochlodon vorosi* is most similar to that of *Zalmoxes robustus* in having a slender shaft, a well-developed proximal articulation with a massive olecranon process. The distal end is flattened mediolaterally, slightly wider dorsoventrally, and slightly curved ventrally relative to the shaft. However, the ulna of *Mochlodon* is proportionally more slender and elongate compared to that of *Z. robustus*. Laterally, just cranial to the olecranon process, the humeral articular facet is developed as a distinct protuberance. Craniodorsally and cranially, a rugose surface represents the articular facet for the proximal radius. In dorsal view, the ulna is very slightly curved medially toward its distal end. The medial surface of the distal end is slightly striated, marking the articular facet for the distal radius. The distal articular facet of the ulna is gently convex.

**Figure 7:**
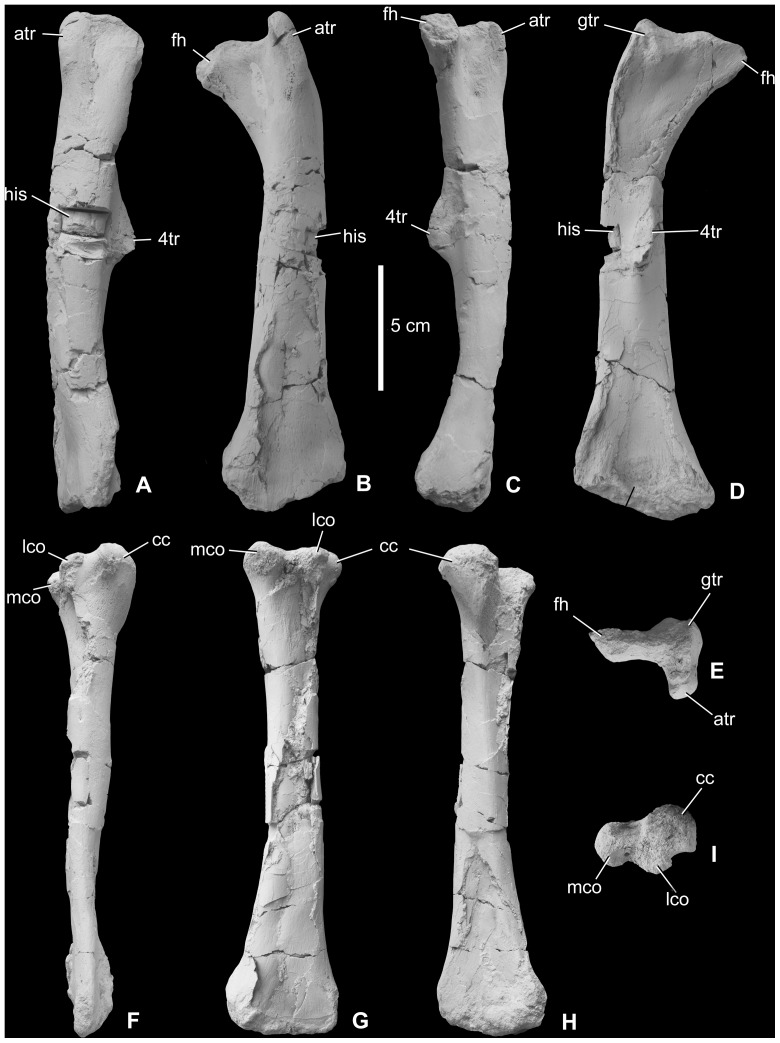
Hindlimb elements of *Mochlodon vorosi* n. sp. from the Upper Cretaceous Csehbánya Formation, Iharkút, western Hungary. A, left femur (MTM V 01.225.) in lateral, B, craniaL, C, medial, D, caudal, E, proximal views; F, right tibia (MTM V 2010.127.1.) in lateral, G, caudal, H, cranial, I, proximal views. Anatomical abbreviations: atr, cranial trochanter; cc, cnemial crest; fh, femoral head; gtr, greater trochanter; his, place of histological sampling; lco, lateral condyle; mco, medial condyle; 4tr, fourth trochanter.

*Femur (* *Figure 7A–E* *).* Two almost complete left femora (MTM V 01.225., V 2010.126.1.) and a fragmentary right femur (MTM 2012.25.1) are known. The largest and most complete left femur and the fragmentary right femur are approximately the same size, with an estimated length of ca. 20 cm. This size corresponds to the smallest size category known for the femur of *Zalmoxes robustus*, and the relationship between femoral length and midshaft diameter for *Mochlodon vorosi* fits well the regression line documented by Weishampel et al. [1]. In cranial or caudal view, the femur has a straight shaft with a subcircular midshaft cross section that is slightly compressed craniocaudally. The medial surface of the femoral shaft is not as bowed as that of *Rhabdodon priscus* or *Z. robustus*. In lateral view, the femur is slightly bowed cranially (Figure 7A). The lateral surface of the femur is straight in cranial view, but at its proximal end it curves slightly medially, more-or-less similar to the femur of *Z. robustus* [1]. The femoral head is eroded so its original shape and medial extension is unknown. The femoral neck is craniocaudally flattened. Ventrally, the neck is continuous with a marked, slightly concave ridge that extends distally along the caudal surface of the shaft; this ridge connects to the proximal part of the prominent fourth trochanter. On the lateral surface of the proximal end, the cranial trochanter is finger-like and separated from the greater trochanter by a narrow groove. Proximally, the greater trochanter has a slightly convex, crest-like lateral surface that becomes saddle-shaped toward the femoral neck. Caudomedially, this surface ends in a marked protuberence. The prominent fourth trochanter becomes higher distally, has an apex that extends 1.2 cm from the shaft, and terminates just at half the length of the bone. Whereas on the fragmentary right femur the fourth trocanter is not pendent and thus is quite similar to that of *Z. robustus*, in the most complete femur it appears that the fourth tranchanter had a small pendent end (Figure 7A, D), although not as strongly developed as in *Hypsilophodon* [51] and other basal ornithopods. In *Mochlodon suessi* (PIUW 2349/3), the fourth trochanter is very similar to that of *M. vorosi*. The bone surface of the most complete femoral specimen is well preserved and shows several muscle attachment areas. Among these, one of the most rugose and irregular is positioned just medial to the fourth trochanter and represents the insertion surface of part of *musculus caudofemoralis*. Distally, the medial and lateral surfaces of the femur diverge strongly from one another in cranial or caudal view. The distal end is not missing in every specimen.

*Tibia (* *Figure 7F–I* *).* One complete right (MTM V 2010.127.1) tibia, the distal two-thirds of a left tibia, and two fragmentary left tibiae (MTM V 01.101., 2012.26.1) are referred here to *Mochlodon vorosi*. The complete right tibia is 142 mm long, but the distal left tibia has an estimated length of 170 mm. The tibia of *M. vorosi* shows multiple characters that are different from those of *Zalmoxes*. First, as also described for most other limb elements, the tibia is much more gracile than that of other rhabdodontids (even more gracile than that of *Z*. *shqiperorum*, [23]) and it is rather similar to the tibia of *Orodromeus makelai* [52]. It is straight and not as strongly bowed in cranial view as that of *Z*. *robustus*. In addition, the proximal and distal ends are not as strongly expanded relative to the shaft as in other rhabdodontids. In the complete specimen, the proximal end is well preserved, showing the almost equal-sized inner and outer condyles that are both directed slightly caudally. They are separated caudally by a deep intercondylar groove. Cranial to the outer condyle is an enormous, rounded cnemial crest, which is twice as large as the other condyles and which is separated from the outer condyle by a deep notch. This massive crest extends distally and merges into the shaft. Laterally, the crest bears a small, pointed, protuberance in proximal view. The articulation surface of the proximal tibia is rugose. Laterally, on the proximal third of the shaft, a small (1.5 mm) foramen is present. The distal half of the shaft is twisted at an angle of 110° relative to the proximal end. The distal end is expanded mediolaterally relative to the shaft, but not as strongly as in *Z*. *robustus*. Whereas on the smaller but complete specimen (V 2010.127.1) the lateral part of the distal end of the tibia (external malleolus) does not extend more distally than the medial part, on the largest specimen the external malleous extends more distally but not to a comparable extent to that seen in *Zalmoxes*.

*Phalanges.* Two well-preserved phalanges (MTM 2012.27.1, 2012.28.1) have been found in Iharkút, which on the basis of size and morphological similarities, are referred here to *Mochlodon vorosi* and they are thought to be pedal phalanges. They are wider than high and possess a concave, oval-shaped proximal articular surface and a well-developed distal articular surface with two distinct condyles separated by an intercondylar groove. These phalanges do not exhibit well-developed dorsal (extensor) processes, similar to *Zalmoxes*.

### Phylogenetic analysis

Analysis of the modified dataset of Weishampel et al. [1] produced two most parsimonious trees with a length of 156 (CI = 0.532, HI = 0.467, RI = 0.771, RC = 0.41). The analysis supports the hypothesis that *Mochlodon vorosi* is a member of Rhabdodontidae: it is recovered as the sister taxon of *Zalmoxes*, and together they form an eastern European lineage that is the sister taxon of the *Rhabdodon* lineage from western Europe. The rhabdodontid clade was placed as the sister taxon to the clade consisting of *Tenontosaurus*, *Dryosaurus*, *Camptosaurus* and *Iguanodon*.

**Figure 8:**
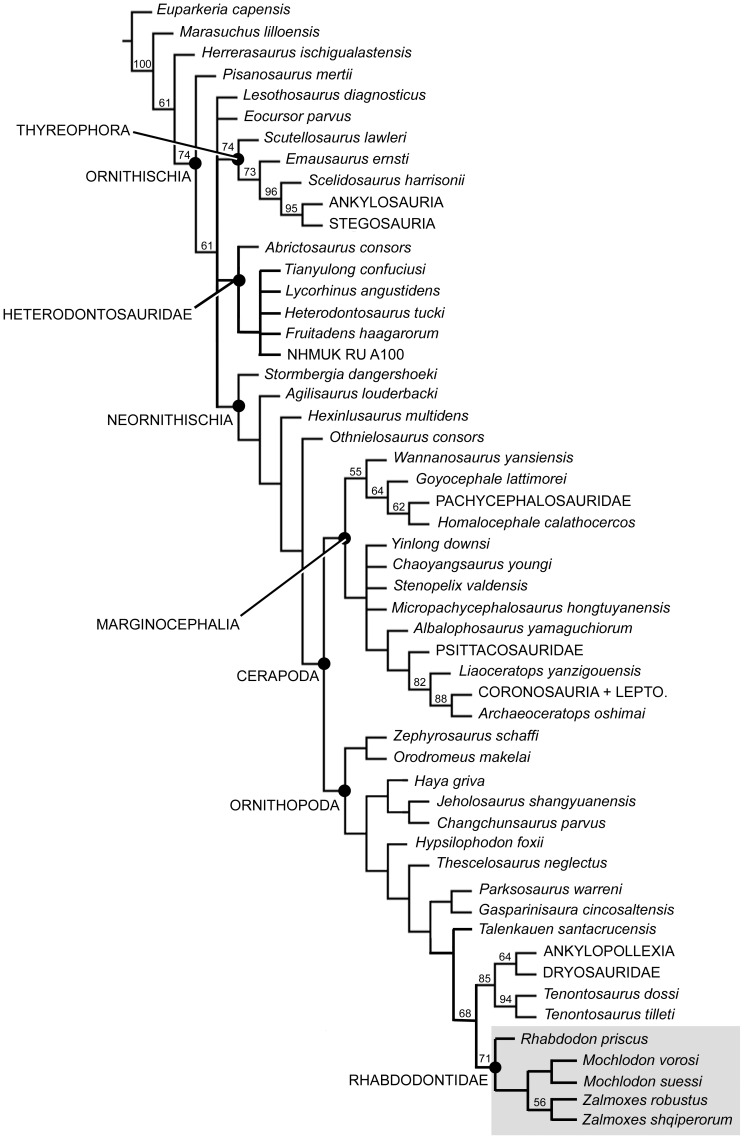
Strict reduced consensus tree of ornithischian interrelationships based upon the reanalysis of the dataset of Han et al. [**39**]. Bootstrap values are shown above branches.

Analysis of the modified dataset of Han et al. [39] recovered 1728 most parsimonious trees (MPTs) of 608 steps. The strict consensus of these trees was poorly resolved. However, a monophyletic Rhabdodontidae was recovered, and included *Rhabdodon priscus* as the sister taxon to a *Mochlodon* + *Zalmoxes* clade. The rhabdodontid clade was placed as the sister taxon to *Tenontosaurus* + (Dryosauridae + Ankylopollexia). A strict reduced consensus tree calculated *a posteriori*, excluding a number of taxa (*Yandusaurus hongheensis*, *Anabisetia*, *Yueosaurus*, and *Koreanosaurus*) previously identified as wildcards by Han et al. [39], shows substantially better resolution (Figure 8) and is used as the basis for subsequent discussion and analysis. Unambiguous synapomorphies were determined using TNT. Rhabdodontidae was supported by the presence of an ischium with a shaft that is gently curved along its length (character 180, state 1), 10 or fewer dentary teeth (character 228, state 0), and dentary crowns with more than 10, and often more than 17 ridges (character 229, state 1). The *Zalmoxes* + *Mochlodon* clade was unambiguously supported by the coracoid having an extremely elongated sternal process (character 231, state 1), *Mochlodon* by the presence of a depression on the caudolateral surface of the dentary (character 230, state 1), and *Zalmoxes* by the presence of a more distally positioned fourth trochanter (character 202, state 1). All of these clades may also be supported by additional characters, but the optimization of many characters is ambiguous due to high amounts of missing data.

### Ontogenetic stages inferred from bone histology, and estimated body sizes

#### *Mochlodon vorosi* (Figure 9, 10)

**Figure 9:**
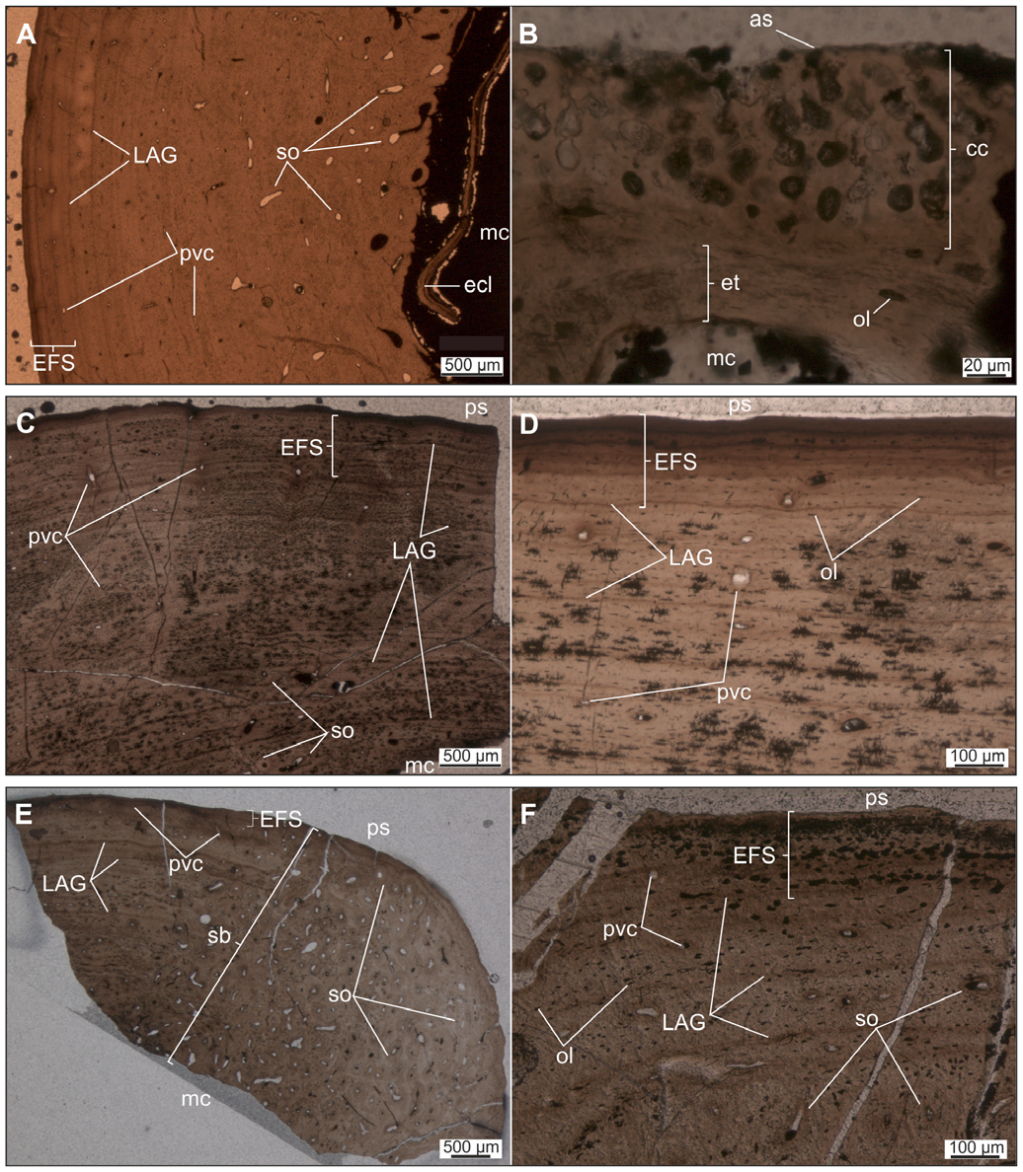
Thin sections of various limb bones of *Mochlodon vorosi* exhibiting adult microstructural features. A–B. Diaphyseal cross section (A), and longitudinal section of the proximal epiphysis (B) of humerus (MTM 2012.23.1). C–D. Diaphyseal cross section of femur MTM V 01.225. E–F. Diaphyseal cross section of tibia MTM V 01.101. Abbreviations: as, articular surface; cc, hypertrophied calcified cartilage; ecl, endosteal circumferential lamellae; EFS, external fundamental system; et, endosteal trabecular bone; LAG, lines of arrested growth; mc, medullar cavity; ol, osteocyte lacunae; ps, periosteal surface; pvc, primary vascular canal; sb, secondary bone; so, secondary osteon.

**Figure 10:**
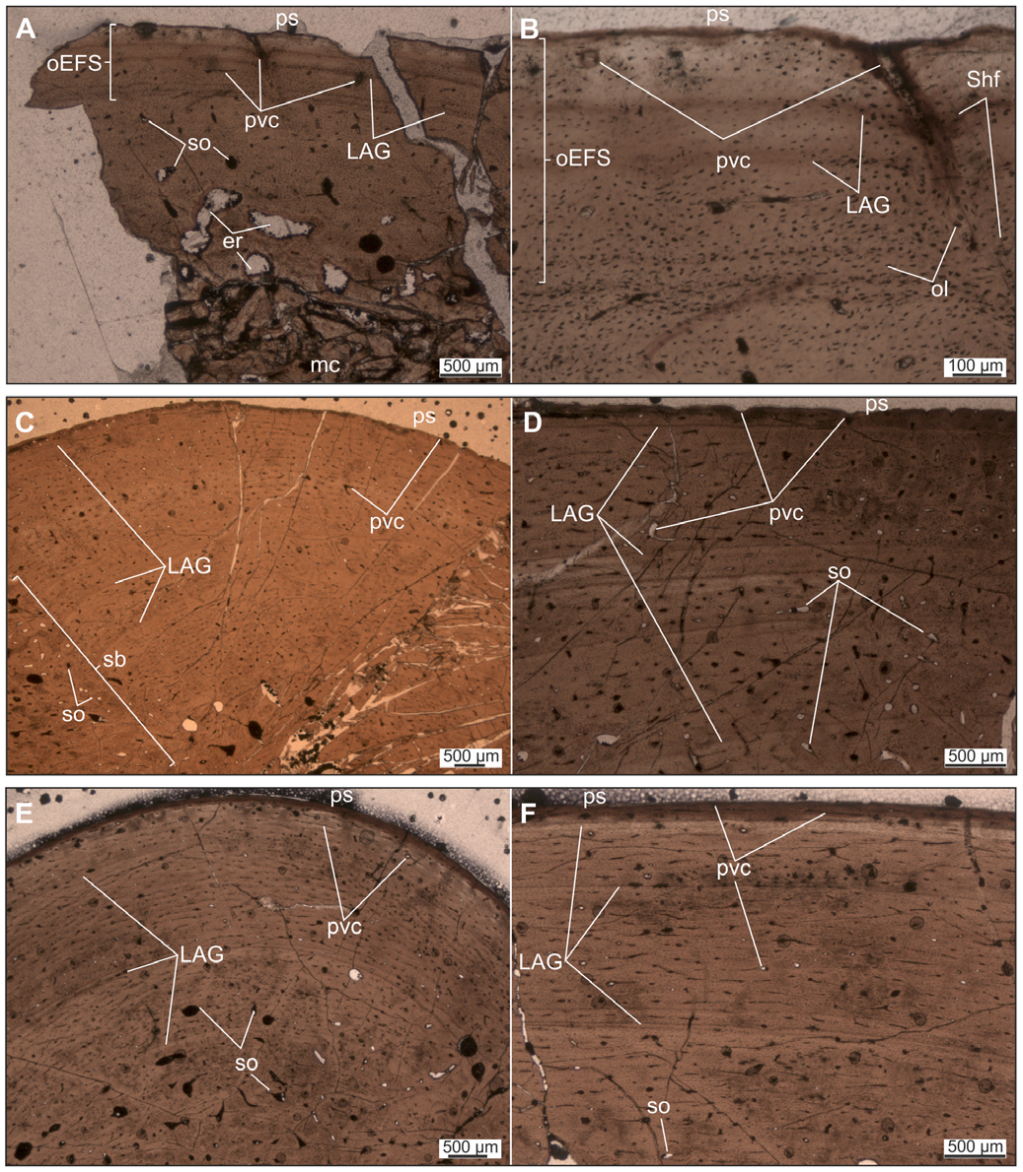
Thin sections of various limb elements of *Mochlodon vorosi* n. sp. A–B. Diaphyseal cross section of femur MTM V 2010.126.1. exhibiting subadult histology. C–D. Diaphyseal cross section of femur (MTM 2012.25.1) showing late juvenile microstructural features. E–F. Diaphyseal cross section of tibia (MTM 2012.26.1). Abbreviations: er, erosion room; oEFS, onset of an EFS; Shf, Sharpey's fibres. For further abbreviations see Figure 9.

The histological features identified in the bones of *Mochlodon vorosi* unequivocally demonstrate the small adult body size of this species. Based on their microstructure, a humerus (MTM 2012.23.1) represent fully grown individuals with estimated body lengthes of only 1.8 m, 1.6 m, and 1.2 m, respectively. The complete cross section of the mid-diaphysis of the humerus (Figure 9A) shows an almost avascular peripheral-most cortical region with increasing number of closely spaced LAGs that represents an EFS (external fundamental system); the histological signal of cessation of growth. The adult stage is also confirmed by the very small and rounded osteocyte lacunae in the majority of the primary cortex. Traces of the EFS are also recognizable in the transverse and longitudinal sections of the proximal epiphysis of this bone. As expected in a skeletally mature animal, no remnants of calcified cartilage are present in the epiphyseal sections, except for a very thin layer on the articular surface that is visible in the longitudinal section (Figure 9B). In the cross-sections of the femur and tibia (Figure 9C–F), there is a pattern of progressively more densely packed LAGs toward the peripheral cortex, and the outermost thin layer is almost avascular. This bone composition also indicates the presence of an EFS. Furthermore, the tibia locally exhibits extensive cortical remodeling (Figure 9E), which is also a characteristic feature of advanced developmental stages.

Among the six investigated bones, only one specimen, a femur (MTM V 2010.126.1), exhibits subadult microstructural features (Figure 10A, B). In this specimen, the outermost primary cortex still contains some vascular canals; however, their number decreases toward the periosteal surface. LAGs also become more frequent and closely spaced peripherally. Although this femur was still capable of diametrical growth to some extent, this pattern indicates the onset of an EFS with a drastic decrease in growth rate. Because the cessation of growth cannot be confirmed, the ontogenetic age of the specimen is defined as subadult. Based on the dimensions of this femur, the estimated total body length for this individual is 1.2 m.

The remaining sampled bones, a femur (MTM 2012.25.1, Figure 10C, D) and a tibia (MTM 2012.26.1, Figure 10E, F), show juvenile histological characteristics, most probably representing late juvenile bones. In contrast to the adult and subadult femora, neither increase in the number of LAGs or lamellar deposition, nor decrease in vascularization, can be observed in the peripheral-most cortical microstructure of the late juvenile femur (Figure 10C, D). Furthermore, the osteocyte lacunae are larger, rounder, and their density also seems to be higher than in the more mature femora. Similar to the juvenile femur, no structural change toward the periosteal surface can be recognised in the tibia, with the exception of a thin layer of diagenetic colour modification in the peripheral-most cortex (Figure 10E, F). The late juvenile status of these two bones is inferred based on both the diameter of the vascular lumina, which are smaller than those characterizing earlier juvenile stages, and the relatively extensive secondary remodeling that took place in the perimedullary region and also locally in the deeper cortex. The estimated length of the femur and tibia resulted in estimated total body lengths of 1.6 m and 1.4 m, respectively. However, these results are surprisingly high for late juveniles, when compared to the estimated adult body lengths based on the histologically mature femur and tibia.

#### *Mochlodon suessi*, *Zalmoxes*, *Rhabdodon* (Figure 11, 12)

**Figure 11:**
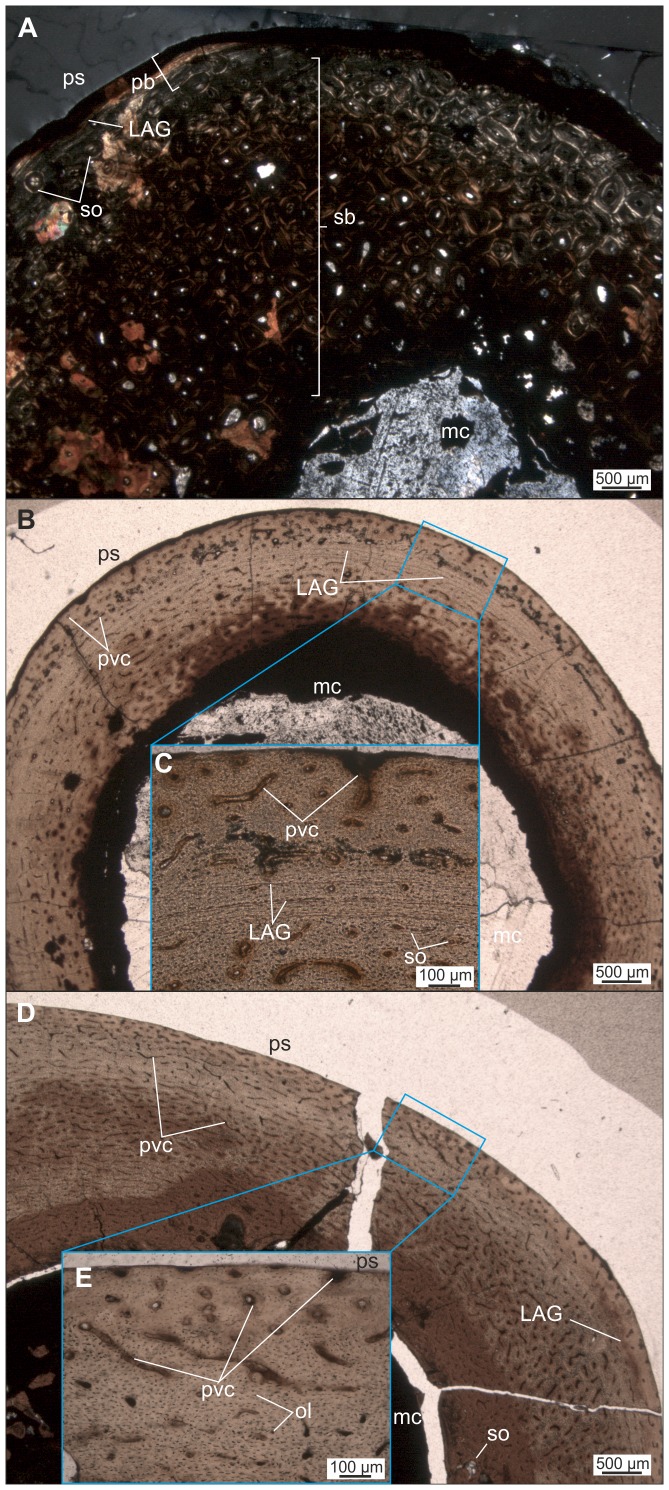
Thin sections of different long bones of *Mochlodon suessi*. Histological features show that both, adult (A) and juvenile (B–E) ontogenetic stages are represented in the sample. A. Diaphyseal cross section of tibia PIUW 2349/35. B–C. Diaphyseal cross section of radius PIUW 3517 with close up (C) of the outermost cortical microsturcture. D–E. Diaphyseal cross section of femur PIUW 2349/III with close up (E) of the outermost cortical microsturcture. Abbreviation: pb, primary bone. For further abbreviations see Figure 9.

**Figure 12:**
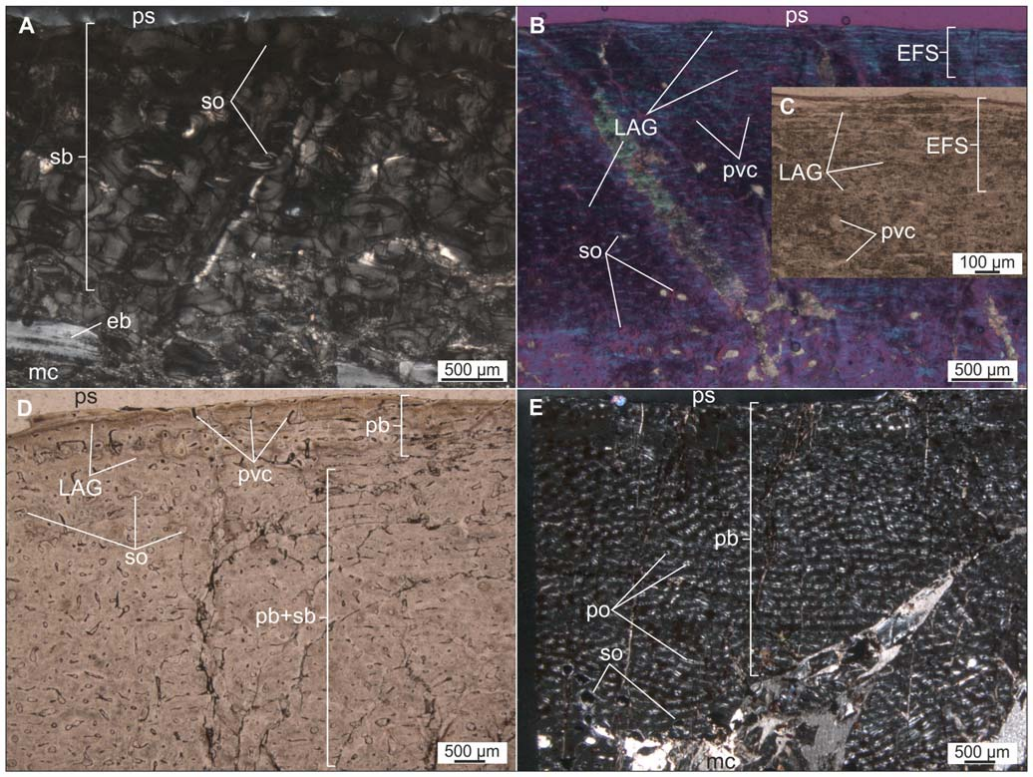
Thin sections of limb bones of *Rhabdodon* sp. Based on bone microstructure, adult (A–C), late juvenile (D) and juvenile (E) ontogenetic stages can be identified. A. Distal metaphyseal cross section of femur MHN AIX PV 2007.4.116. B–C. Mid-diaphyseal cross section of femur MHN AIX PV 2008.1.11 with close up (C) of the outermost cortical microsturcture. D. Proximal diaphyseal cross section of humerus Mechin coll. 472. E. Mid-diaphyseal cross section of humerus MHN AIX PV 2001.65. Abbreviations: eb, endosteal bone; pb+sb, primary bone invaded by secondary osteons. For further abbreviations see Figure 9 and 11.

The four sampled postcranial elements of *Mochlodon suessi* represent mainly histologically immature individuals (Figure 11B–E) with a body length ranging from 0.8 to 1.6 m. Only one element, a tibia (PIUW 2349/35), exhibits microstructural features implying an adult ontogenetic stage (Figure 11A). The body length of this adult individual is inferred to have been about 1.4 m, closely matching the subadult–adult size range of *Mochlodon vorosi*. However, similar to the condition in *M. vorosi*, a 1.6 m long late juvenile was identified that is relatively large compared to adult specimen with an inferred body length of 1.4 m.

Sampled bones of *Zalmoxes* included elements referred to the two named species, *Z. robustus* and *Z. shqiperorum*, as well as specifically indeterminate specimens (*Zalmoxes* sp.). *Z. robustus* is represented by a late juvenile of 2.3 m body length and two subadults with estimated body lengths of 2 m and 2.4 m. The two sampled bones assigned to *Z. shqiperorum* indicate the presence of a juvenile of 1.2 m and a subadult of 2.5 m body length. Inclusion of the results obtained from the two sampled bones of *Zalmoxes* sp. appears to extend the range of possible adult body sizes. One of the two sampled humeri, belonging to an individual with an estimated body length of 2 m, already shows subadult histological characteristics, whereas the other humerus, which is inferred to have belonged to an animal of approximately 2.9 m length, exhibits microstructural features of a late juvenile.

The sampled bones of *Rhabdodon* (Figure 12) all represent specimens of as yet undetermined specific affinities (*Rhabdodon* sp.). The inferred body sizes of individual ontogenetic stages show substantial variation. Body length estimates of juveniles (Figure 12D, E) and late juveniles range from 2.7–5.1 m, whereas, based on the two elements that show mature bone microstructure, two different individuals achieved adult body sizes at total body lengths of 5.9 m (Figure 12A) and 1.5 m (Figure 12B, C).

**Figure 13:**
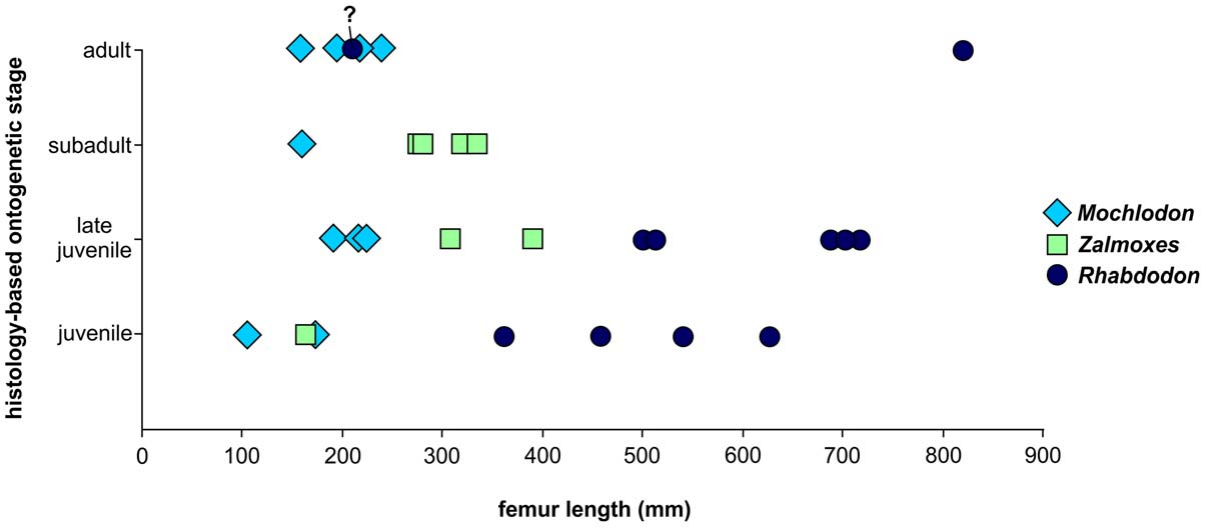
Plot of femoral length vs. histological ontogenetic stage of all histologically sampled specimens. Specimens of *Mochlodon* (blue diamond), *Zalmoxes* (green square) and *Rhabdodon* (dark blue circle) are indicated at genus level. Note the single outlier datapoint of an adult *Rhabdodon* specimen (labelled with question mark) which fits the femoral length range represented by adult *Mochlodon* species.

Our comparative histological study of all rhabdodontid genera known from Europe, with special emphasis on *Mochlodon vorosi*, demonstrated similar overall bone tissue characteristics for each taxon, implying similar growth rates in these closely related groups. However, the typical histological features of particular ontogenetic stages are manifested at markedly different body sizes across the sampled taxa (Figure 13), indicating substantial variation in body size within Rhabdodontidae. More details on the specimens sampled for histological investigation are given in Table 1.

### Body size evolution

**Figure 14:**
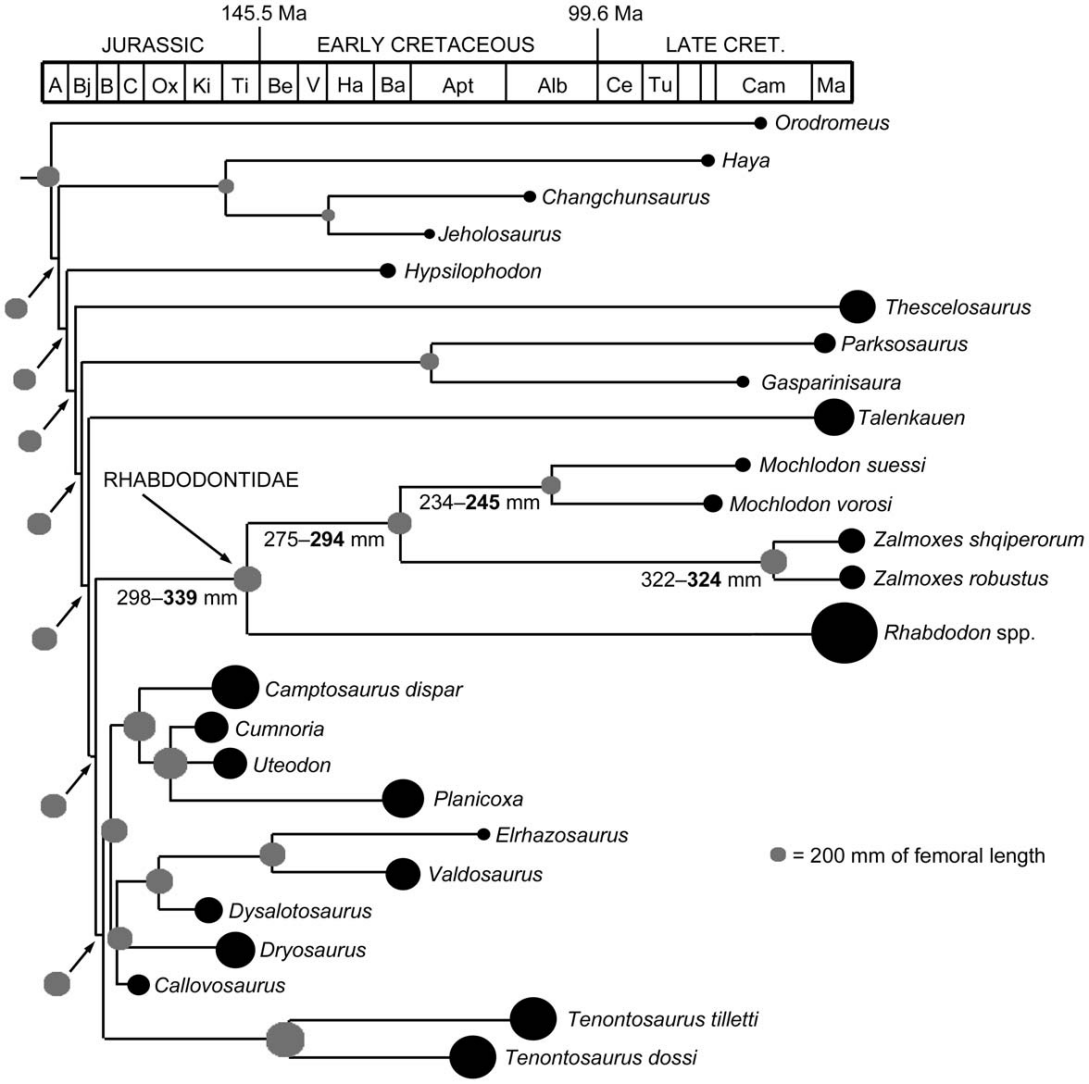
Quantitative reconstruction of body size evolution among non-hadrosauriform ornithopods. Black circles represent body size (based upon femoral length) of terminal taxa, whereas grey circles indicate reconstructed ancestral body sizes. Within the clade Rhabdodontidae two values for femoral length are reconstructed at each ancestral node: values in bold were calculated using the topology shown here, whereas non-bold values were reconstructed using a topology in which *Rhabdodon* was split into giant and small species.

The analysis reconstructed (Figure 14) moderately sized (280–340 mm) ancestral femoral lengths along much of the backbone of basal ornithopod phylogeny, with very small ancestral femoral lengths reconstructed only close to the very base of the clade. A femoral length of approximately 339 mm is reconstructed as the ancestral state for Rhabdodontidae, very close in size to the femoral lengths known for *Zalmoxes* (maximum femoral lengths of 320–333 mm used in this study). Thus, this analysis does not support the hypothesis of autapomorphic nanism (‘island dwarfism’) in *Zalmoxes* [26], which assumes that this genus is significantly smaller relative to the plesiomorphic condition for the rhabdodontid clade. *Rhabdodon* is reconstructed as having undergone autapomorphic giantism and *Mochlodon* is reconstructed as having an ancestral femoral length of 245 mm, thus potentially consistent with a reduction in size relative to the ancestral rhabdodontid condition (ca. 340 mm). Inclusion of small and large sister taxa within *Rhabdodon* results in a smaller ancestral femoral length (298 mm) for Rhabdodontidae, also failing to support the hypothesis of autapomorphic nanism for *Zalmoxes*.

## Discussion

### Temporal and spatial distribution of Rhabdodontidae

Rhabdodontidae is a relatively well-supported family-level clade of ornithopod dinosaurs that is endemic to Europe ([1], [27], this paper). At the present, the clade includes six species referred to three genera: *Rhabdodon priscus* and *R. septimanicus*, *Mochlodon suessi* and *M. vorosi*, and *Zalmoxes robustus* and *Z. shqiperorum*. In addition, the ‘Villeveyrac rhabdodontid’ from the early Campanian of France may represent a third species of *Rhabdodon* [53]. Whatever the phylogenetic position of this undescribed rhabdodontid proves to be, it likely is the oldest known representative of *Rhabdodon* and thus may play a critical role in a better understanding of the origin, evolution and distribution of the clade. The currently known temporal range of Rhabdodontidae is approximately 15–18 million years, bracketed by the oldest form, *Mochlodon vorosi* from the Santonian of Hungary (described here), and *Zalmoxes* spp. from the Maastrichtian of Romania [1], [27], [54], [55]. The known range of *Rhabdodon* (including the Villeveyrac form, [53]) extends at least from the early Campanian to the early Maastrichtian [10].

Combining the results of the phylogenetic analyses with current understanding of Late Cretaceous palaeobiogeography, we propose that at least two major lineages of the rhabdodontid clade existed within separate parts of the Late Cretaceous archipelago of the western Tethys. The western lineage (France, Spain) is represented by *Rhabdodon* and its ancestors. The temporal range (a minimum of 10–12 million years) of the rhabdodontid material in western Europe, along with the markedly different sizes of adult specimens, suggests that rhabdodontid ornithopods were likely more diverse in western Europe than previously thought, with at least three and perhaps even four different species (*R*. *priscus*, *R*. *septimanicus*, the Villeveyrac rhabdodontid, and the small form represented by MHN AIX PV 2008.1.11). The eastern lineage (Austria, Hungary, Romania) is represented by *Mochlodon*, *Zalmoxes* and their ancestors. This lineage is represented by least with four different species (*M*. *suessi*, *M*. *vorosi*, *Z*. *robustus*, *Z*. *shqiperorum*). The common ancestor of the western and eastern lineages must pre-date the Santonian. However, the pre-Santonian Late Cretaceous terrestrial fossil record is very poorly known in Europe, and this is particularly true for ornithopods. Chronologically and geographically, the closest pre-Santonian records to *Mochlodon vorosi* are some isolated bones and teeth from the Cenomanian of Czech Republic [56], western France [57], that have been identified as *Iguanodon*-like ornithopods. In addition a tooth from the Cenomanian of England has been referred to *‘Iguanodon hilli*’ [58] that is regarded as nomen dubium by Horner et al [59]. However, these Cenomanian forms likely belong to a clade of iguanodontian ornithopods more closely related to hadrosaurids than to Rhabdodontidae. Thus the pre-Santonian evolutionary history of the rhabdodontid clade remains unsampled, and known members of Rhabdodontidae are separated by a ghost lineage of at least 75 million years from their closest relatives within Ornithopoda (see also [27]).

### Implications based on ontogenetic stages vs. estimated body lengths within Rhabdodontidae

**Figure 15:**
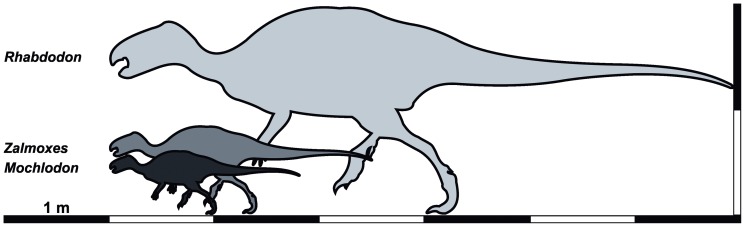
Comparison of histology-based adult body sizes of *Mochlodon*, *Zalmoxes* and *Rhabdodon* represented by the silhouettes of the animals.

Whereas no substantial difference was observed in the overall patterns of bone tissue types deposited in the bones of different members of Rhabdodontidae, variability in the body sizes of particular ontogenetic stages delineates distinct groups between and within the sampled taxa (Figure 15). Although the relatively small sample size considered here precludes statistical analysis, histological analysis of all currently recognised genera within Rhabdodontidae provides insights into evolutionary changes in adult body sizes within this clade. These results have further important evolutionary implications when considered within the context of the temporal and spatial distribution of rhabdodontids within the Late Cretaceous western Tethyan archipelago. Although it has been demonstrated that the phylogenetic signal contained within bone histological features in sauropsids is slight or non-significant [60], considerable differences in the adult body sizes recognised in this study may have important implications for previous and future taxonomical assignments.

The two species of *Mochlodon*, *M. vorosi* and *M. suessi* could not be distinguished on the basis of body length estimates inferred from histological data. Corresponding ontogenetic stages occur at closely similar body size ranges. These findings support the taxonomic results presented here, which reintroduce and validate the name, *Mochlodon*, for these two species.

*Zalmoxes* and *Mochlodon*, however, exhibit discernible size differences at equivalent ontogenetic stages. Whereas an estimated body length of 2 m is the lowest value known for late juveniles of *Zalmoxes*, the two species of *Mochlodon*, *M. vorosi* and *M. suessi*, already show adult microstructural features at body lengths of 1.2–1.8 m. These results imply that the final adult body size of both *Mochlodon* species was notably smaller than that of any known species of *Zalmoxes*. Nevertheless, the inferred body size of the largest adult specimen of *Zalmoxes* (FGGUB R.1608) also does not exceed 2.5 m in length (Figure 13).

As in *Mochlodon*, the two known species of *Zalmoxes*, *Z. robustus* and *Z. shqiperorum*, cannot be distinguished based on the estimated body lengths of the histologically sampled specimens [26]. When data acquired from specifically undetermined *Zalmoxes* bones are also included, the range of actual adult body sizes appears to increase. The size deviation demonstrated by subadults of 2 m and a late juvenile of 2.9 m body length may indicate intraspecific variability or could alternatively reflect taxonomic difference.

The estimated body length data for the sampled specimens of *Rhabdodon* are very hard to interpret, assuming the presence of only a single species. Differences in body size occurring within a single ontogenetic stage are so pronounced that it seems more likely that they indicate the presence of at least two, but perhaps multiple, taxa. The recognition of at least two taxa is based on the huge size difference between the two recognised adult specimens, with one individual having an estimated body length of 5.9 m (MHN AIX PV 2007.4.116) and another only 1.5 m body length (MHN AIX PV 2008.1.11, Figure 13). Based upon the general intraspecific body size distribution throughout ontogeny of extant endotherms, three size groups appear to be present on a finer scale within the *Rhabdodon* material. Nevertheless, each group lacks histologically-demonstrable representatives of one or more ontogenetic stages. The first group is represented by juveniles, late juveniles and an adult with body length ranges of 4–4.5 m, 4.9–5.1 m, and 5.9 m, respectively. The next size group consists of two late juveniles estimated at body lengths of 3.7 m, suggesting that the adults of this size group presumably did not exceed 4.5–5 m. Two additional juvenile specimens with inferred body lengths of 2.7 and 3.4 m could equally belong to either the first or the second size group. The third, most distinct, size-group is represented by a single femur of an individual with an estimated body length of only 1.5 m. This specimen unequivocally exhibits mature histological features that reveal a final body size that is definitely an extreme outlier within *Rhabdodon*, but is well within the range of both *Mochlodon* species.

Nonetheless, if considerable intraspecific variability in body sizes is presumed, the first two size groups could be united into a single developmental series. In this case, the observed diversity in realized body sizes within a single ontogenetic stage could be explained by two different phenomena. First, sexual dimorphism could be expressed by significantly different body sizes between the two sexes: i.e. the first and second size groups identified here. In this case, a bimodal size distribution would be expected with a larger, statistically analysable, dataset. Alternatively, intraspecific size deviations of this extent might be the result of developmental plasticity; a phenomenon already suggested for *Plateosaurus* [61], and also for the pterosaur *Rhamphorhynchus* [62]. Even if intraspecific variability of either kind accounts for most of the revealed diversity in the growth trajectories of different individuals, the extremely small mature specimen, representing by itself the third size group, cannot be incorporated into the developmental series represented by the remaining specimens. Thus, the most parsimonious interpretation is the assignment of this specimen to a different, probably as yet unrecognised, species.

### Body size evolution in Rhabdodontidae: giantism vs. nanism?

Ever since it was first proposed [63], the hypothesis that ‘insular dwarfism’ characterised the Transylvanian dinosaurs has intrigued scientists, and became widely supported following the work of Benton et al. [26]. Using histological features, Benton et al. [26] suggested that the largest available (although still subadult) specimens of *Zalmoxes* were significantly smaller than closely related ornithopods such as *Tenontosaurus*, *Camptosaurus* or *Rhabdodon*. Because animals histologically defined as subadults supposedly do not grow much further they concluded that, based on the observed size differences, *Zalmoxes* was probably an island dwarf, similar to the contemporary hadrosauroid *Telmatosaurus*, both of which inhabited the Maastrichtian Haţeg Island. However, when comparing body sizes, Benton et al. [26] restricted the phylogenetic context of *Zalmoxes* to a few bracketing taxa, and did not perform any quantitative analysis of body size evolution. Body size measures mapped on a broader, more detailed phylogenetic tree would have been necessary to support their view on the island dwarfism of *Zalmoxes*, i.e. a form of autapomorphic nanism [35]. Posing the same question, Weishampel and Jianu [27] included more taxa in their numerical analysis to explore the evolutionary trends in body sizes of euornithopods with special focus on *Zalmoxes*. Based on their results, they concluded that there is indeed a peramorphocline traceable from basal euornithopods (such as *Orodromeus*) to more derived taxa (like *Tenontosaurus*), and within this pattern they recognized an autapomorphic size decrease in *Zalmoxes robustus*. Thus Weishampel and Jianu [27] regarded *Zalmoxes robustus* as a potential dwarf and *Z. shqiperorum* as peramorphic in keeping with the general ornithopod trend.

**Figure 16:**
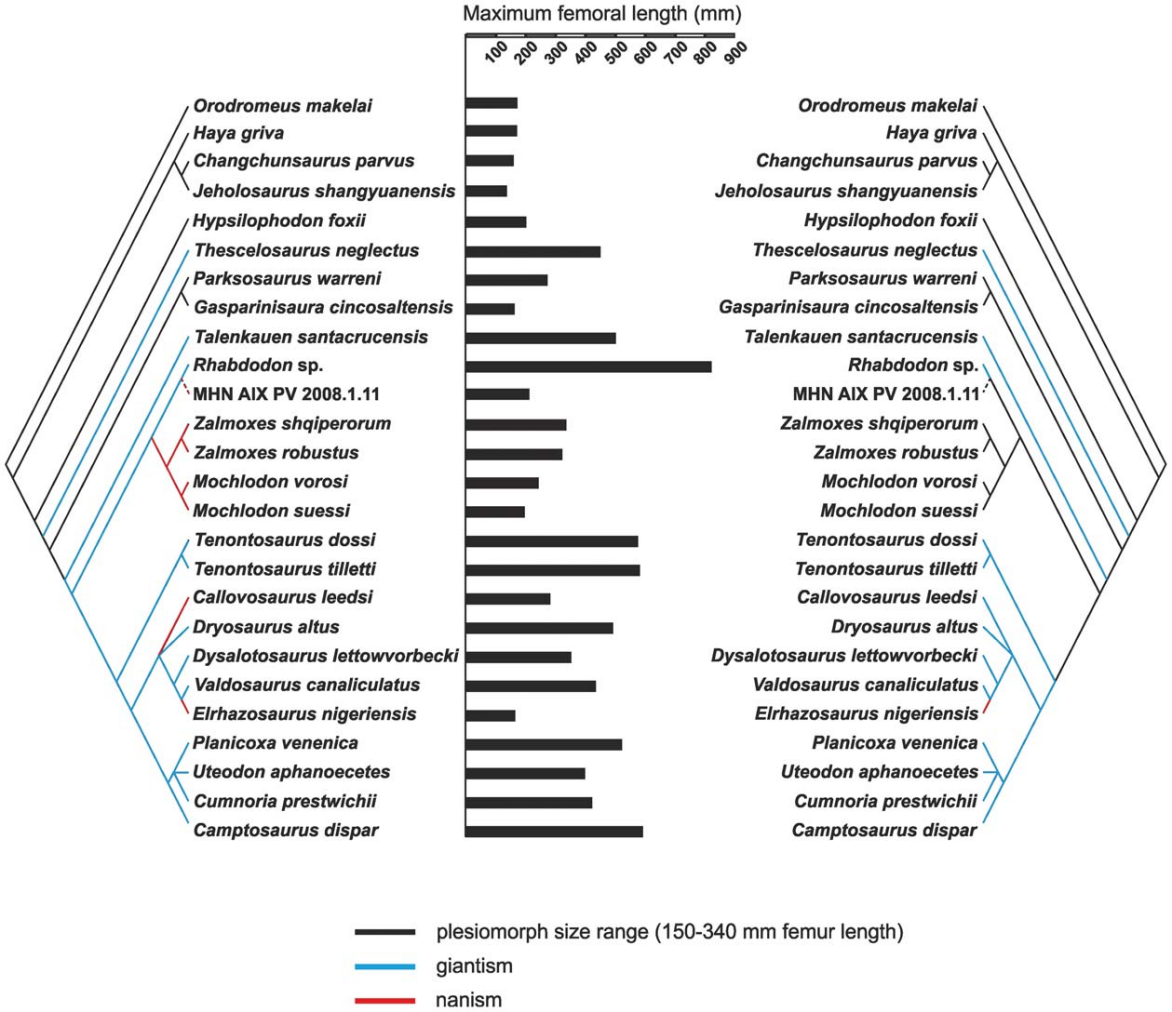
Contrasting two of the numerous possible interpretations on body size evolution in basal ornithopods from *Orodromeus* up to *Camptosaurus*. Plesiomorphic body size (302–340 mm femur length, black line) and presumed phyletic and/or autapomorphic giantism (blue line) and/or nanism (red line) are indicated on the branches of the tree with special focus on the clade Rhabdodontidae. The tree on the left arbitrarily infers phyletic giantism from the branch of *Talenkauen* on, thus on the ghost-line leading to Rhabdodontidae too. In this case, only *Rhabdodon* follows this trend, whereas for all other members of the clade (*Zalmoxes* and *Mochlodon* species) phyletic or autapomorphic nanism must be presumed. In contrast, the tree on the right demonstrates that the ancestral condition is retained throughout the whole clade except for *Rhabdodon*, which has undergone autapomorhic giantism. With respect to the clade Rhabdodontidae, the latter interpretation gained more support by our numerical analysis.

Our investigation shows that the final body size of both species of *Mochlodon* is even smaller than that of their closest relative, *Zalmoxes*. Since both genera belonged to an island fauna, the need for reexamination of the island dwarf hypothesis is evident when *Mochlodon* is considered. The results of the numerical analysis testing for both autapomorphic as well as phyletic changes of body size within Rhabdodontidae demonstrated that body size of *Zalmoxes* (with femoral length of 320–333 mm) was not smaller than the reconstructed ancestral condition for the clade (femur length of 298–339 mm), thereby challenging the hypothesis that the members of this genus represent island dwarfs (Figure 16). In fact, *Rhabdodon* appears to be an autapomorphic giant within Rhabdodontidae (with up to 820 mm femoral length, Figure 16), and both species of *Mochlodon* seem to be characterized by autapomorphic nanism (with 234–245 mm ancestral femur length); however, the body size of the latter also does not differ substantially from the inferred ancestral condition. Because no decrease in body size is reconstructed on the branch leading *Zalmoxes* and because only a slight decrease can be demonstrated for *Mochlodon* when compared to the reconstructed ancestral state (Figures 14, 16), phyletic nanism of the *Mochlodon*–*Zalmoxes* clade is not well supported. Although the assumption that small-bodied dinosaurs in the faunas of the Late Cretaceous archipelago of Europe represent island dwarfs has become deeply entrenched, the results of the current study shows that more caution should be taken in assessing the evidence even in apparently ‘unequivocal’ cases. The complex nature of body size evolution and, as a result, the multiple possible ways in which evolutionary patterns can be interpreted have also been demonstrated by Gould and MacFadden [35]. These authors revised widely accepted hypotheses using two case examples (extant members of Varanidae and fossil members of Equidae) with well-known phylogenies that have frequently been cited as showing definite evidence of island giantism (varanids) and Cope's rule (fossil horses). Gould and MacFadden [35] showed that the diversity in final body sizes within the former clades is very high, without revealing any kind of phyletic tendency toward smaller or larger forms. In Varanidae, the difference in maximum body lengths may reach 50% between the two sister taxa, or even between different individuals of the same species [64]. Isolation effects (geographical, ecological, behavioral etc.) do not always provide unambiguous explanations for apparent size increases or decreases in certain branches [35]. A further methodological problem is the inconsistent measurement of size between different studies, which complicates or prevents large-scale comparisons [35], and the fact that no percentage value of size decrease or increase detected between sister taxa can be established from which nanism or giantism could unequivocally be defined for a clade.

Although true phyletic giants (e.g. sauropods: [65]) and island dwarfs (e.g. endemic dwarf elephants of the Mediterranean islands: [66]) do occur among fossil taxa, elucidating tendencies in body size evolution generally proves even more difficult in extinct animals for various reasons. First of all, there is no well-established information on the phylogenetic, ecological and various other supra- as well as intra-individual biological factors, all of which may affect final body size. Uncertainties in taxonomical assignment of fragmentary material and the diverse possible interpretations of phylogenetic relationships have significant influence on the outcome of the numerical analysis. In addition, there is usually no sufficient histological data to support the adult nature of the specimens of terminal taxa on which the numerical analysis of body size evolution is based. In other words, there is no evidence that the specimens, the size of which were used in the reconstruction of ancestral body sizes, were not able to grow any further. The presumably high number of still unknown members of the fossil clade in question presents further difficulties. For instance, the ghost-lineage leading to the oldest known member of Rhabdodontidae represents at least 80 million years (Figure 14), which is much more than enough time for autapomorphic giantism or nanism to occur on multiple occasions. Rapid morphological evolution has been demonstrated for island mammals [67], and this probably holds for other members of island faunas too, rendering reconstructions of the evolution of body size even more difficult. These complications lead to several possible interpretations of how body size could have changed during the evolution of Rhabdodontidae (Figure 16).

Based on our results, autapomorphic size decrease induced by the isolating effect of a true island cannot be excluded in the case of *Mochlodon*, but is unlikely for *Zalmoxes* (see also [27]). The estimated body sizes of the sampled adults of *Rhabdodon* indicates the presence of at least two taxa, one of which approximates to the size range of *Mochlodon*, whereas the other represents a true (probably autapomorphic) giant when compared to all other known rhabdodontid genera and the reconstructed ancestral condition. However, considering all the difficulties related to the reconstruction of body size evolution, our numerical analysis remains inconclusive.

## Conclusions

Remains of a new ornithopod dinosaur have been discovered from the Upper Cretaceous (Santonian) continental deposits of the Csehbánya Formation of Iharkút, western Hungary. Isolated cranial and postcranial remains unambiguously show a rhabdodontid affinity that is strongly supported by a global phylogenetic analysis of ornithischian dinosaurs including all known rhabdodontid genera. The Hungarian form, being the oldest representative of the clade, extends the temporal range (which is approximately 15 million years in total) of the clade from the Santonian to Maastrichtian. Based on characters of the dentary, an element that is known in all rhabdodontid species, the Hungarian species is most similar to the Austrian rhabdodontid. Thus, we resurrected the name *Mochlodon* for the Austrian and Hungarian material but distinguish two different species based upon osteological differences: *Mochlodon suessi* for the early Campanian type material and *Mochlodon vorosi* n. sp. for the Santonian Hungarian remains. This close affinity is further supported by their close temporal as well as spatial proximity. The Hungarian rhabdodontid also shows similarities to *Zalmoxes*, a genus that is approximately 15 million years younger in age, but the morphologies of the quadrate, dentary and some limb bones show important differences between them.

Histological study of limb bones provides reliable estimation of adult body size for all genera of Rhabdodontidae. We concluded that both the Hungarian and Austrian species (*Mochlodon* spp.) were characterized by an adult body length of 1.6–1.8 m that is in accordance with the morphological similarities between these two rhabdodontids. Whereas the subadults of both *Zalmoxes* species were slightly larger (2–2.5 m) than *Mochlodon*, the French specimens of *Rhabdodon* had a much larger, 5–6 m adult body length, indicating a substantial difference in body size between the western and eastern European taxa.

Phylogenetic mapping of femoral lengths onto the results of the phylogenetic analysis reconstructed moderately sized (280–340 mm) ancestral femoral lengths along much of the backbone of basal ornithopod phylogeny and suggests a femoral length close to 340 mm as the ancestral state for Rhabdodontidae, which is very close in size to the femoral lengths of the sampled subadults of both *Zalmoxes* species (320–333 mm). Thus, this analysis does not support the hypothesis of autapomorhic nanism (island dwarfism) in *Zalmoxes* (contra to Benton et al. [26] Weishampel and Jianu, [27]). On the other hand, the 820 mm femoral length of adult *Rhabdodon* sp. specimens demonstrates this genus as an autapomorphic giant within Rhabdodontidae. Although both species of *Mochlodon* (with 159–218 mm adult femur lengths) seem to be characterized by autapomorphic nanism (compared to the 234–245 mm ancestral femur length), their body size does not differ substantially from the inferred ancestral condition. Because no decrease in body size is reconstructed on the branch leading *Zalmoxes* and because only a slight decrease can be demonstrated for *Mochlodon* when compared to the reconstructed ancestral state, phyletic nanism of the *Mochlodon*–*Zalmoxes* clade is not well supported.

These results imply a deep divergence (prior to the Santonian) between a western rhabdodontid lineage represented by at least two species of *Rhabdodon* in Spain and France and an eastern lineage consisting of the *Zalmoxes* and *Mochlodon*.

## Supporting Information

Supporting Information S1 **Additional characters to the Weishampel et al.** [**1**] **matrix.**
(DOC)

Supporting Information S2 **Character matrix of Weishampel et al.** [**1**] **with four new characters listed in Supporting Information S1.**
(TXT)

Supporting Information S3 **Character list of Han et al.** [**39**] **with seven new characters (two of them were also included in the first analysis described above, these are characters 232, 233).**
(DOC)

Supporting Information S4 **Character matrix of Han et al.** [**39**] **with seven new characters (two of them were also included in the first analysis described above, these are characters 232, 233).**
(TXT)
