# Supplementary material for: Phylogeny, Histology and Inferred Body Size Evolution in a New Rhabdodontid Dinosaur from the Late Cretaceous of Hungary
Source: PLoS One. 2012 Sep 21;7(9):e44318. doi: 10.1371/journal.pone.0044318 (PMC3448614; doi:10.1371/journal.pone.0044318)
Supplement: Supporting Information S1 — Additional characters to the Weishampel et al. [1] matrix. (DOC) [file pone.0044318.s001.doc]

**Appendix 1.** Additional charaters to the Weishampel et al. [1] matrix:

76. Distal end of ulna. Primitively, the distal end of the ulna is slightly widens and it is straight or bowed slightly dorsally (0). In *Mochlodon* and *Zalmoxes* the distal ulna is lateromedially flattened and slightly bowed ventrally (1).

77. Dentary symphysis. Basally, the rostral end (symphyseal contact with the predentary) of the dentary curves ventrally and in most cases it curves medially and the rostralmost point of the dentary is well below the level of the alveolar groove (0). In *Mochlodon*, however, this region is straight, medially and ventrally not curved and the rostral most point is in the level of the alevolar groove (1).

78. Quadrate condyles. Primitively, the condyles on the distal quadrate are only slightly enlarged compared to the quadrate shaft, they are notextended rostrocaudally (0). In *Zalmoxes* *robustus*, the distal quadrate condyles are large and rostrocaudally extended (1).

79. Curvature of proximal third of humerus in rostrocaudal view. Primitively, the proximal third of the humerus is slightly bowed medially and the lateral side of the complete shaft of the humerus is more or less straight (0). In more derived forms, the proximal third of humerus is markedly curved medially with an angular lateral corner at the ventral most point of the deltopectoral crest (1).
