# Supplementary material for: Phylogeny, Histology and Inferred Body Size Evolution in a New Rhabdodontid Dinosaur from the Late Cretaceous of Hungary
Source: PLoS One. 2012 Sep 21;7(9):e44318. doi: 10.1371/journal.pone.0044318 (PMC3448614; doi:10.1371/journal.pone.0044318)
Supplement: Supporting Information S3 — Character list of Han et al. [39] with seven new characters (two of them were also included in the first analysis described above, these are characters 232, 233). (DOC) [file pone.0044318.s003.doc]

**Appendix X.**

**Character list of the second analysis**

1. Skull proportions: 0. Preorbital skull length more than 45 per cent of basal skull length; Preorbital length less than 40 per cent of basal skull length.

2. Skull length (rostral–quadrate): 0. 10 per cent or less of body length; 1. 13 per cent or more of body length (modified following Xu *et al*. 2006).

3. Neomorphic rostral bone, anterior to premaxilla: 0. Absent; 1. Present.

4. Rostral bone, anteriorly keeled and ventrally pointed: 0. Absent; 1. Present.

5. Rostral bone, ventrolateral processes: 0. Rudimentary; 1. Well-developed.

6. Premaxilla, edentulous anterior region: 0. Absent, first premaxillary tooth is positioned adjacent to the symphysis; 1. Present, first premaxillary tooth is inset the width of one or more crowns.

7. Premaxilla, posterolateral process, length: 0. Does not contact lacrimal; 1. Contacts the lacrimal, excludes maxilla–nasal contact.

8. Oral margin of the premaxilla: 0. Narial portion of the body of the premaxilla slopes steeply from the external naris to the oral margin; 1. Ventral premaxilla flares laterally to form a partial floor of the narial fossa.

9. Position of the ventral (oral) margin of the premaxilla: 0. Level with the maxillary tooth row; 1. Deflected ventral to maxillary tooth row.

10. Premaxillary foramen: 0. Absent; 1. Present.

11. Premaxillary palate: 0. Strongly arched, forming a deep, concave palate; 1. Horizontal or only gently arched.

12. Overlap of the dorsal process of the premaxilla onto the nasal: 0. Present; 1. Absent.

13. Fossa-like depression positioned on the premaxilla–maxilla boundary: 0. Absent; 1. Present.

14. Premaxilla–maxilla diastema: 0. Absent, maxillary teeth continue to anterior end of maxilla; 1. Present, substantial diastema of at least one crowns length between maxillary and premaxillary teeth.

15. Form of diastema; 0. Flat; 1. Arched ‘subnarial gap’ between the premaxilla and maxilla.

16. Narial fossa surrounding external nares on lateral surface of premaxilla, position of ventral margin of fossa relative to the ventral margin of the premaxilla: 0. Closely approaches the ventral margin of the premaxilla; 1. Separated by a broad flat margin from the ventral margin of the premaxilla

17. External nares, position of the ventral margin: 0. Below the ventral margin of the orbits; 1. Above the ventral margin of the orbits.

18. External naris size: 0. Small, entirely overlies the premaxilla; 1. Enlarged, extends posteriorly to overlie the maxilla.

19. Deep elliptic fossa present along sutural line of the nasals: 0. Absent; 1. Present.

20. Internal antorbital fenestra size: 0. Large, generally at least 15 per cent of the skull length; 1. Very much reduced, less than 10 per cent of skull length, or absent.

21. External antorbital fenestra: 0. Present; 1. Absent.

22. External antorbital fenestra, shape: 0. Triangular; 1. Oval or circular.

23. Additional opening(s) anteriorly within the antorbital fossa: 0. Absent; 1. Present.

24. Maxilla, prominent anterolateral boss articulates with the medial premaxilla: 0. Absent; 1. Present.

25. Maxilla, accessory anterior process: 0. Absent; 1. Present.

26. Maxilla, buccal emargination: 0. Absent; 1. Present.

27. Eminence on the rim of the buccal emargination of the maxilla near the junction with the jugal: 0. Absent; 1. Present.

28. Slot in maxilla for lacrimal: 0. Absent; 1. Present.

29. Accessory ossification(s) in the orbit (palpebral/supraorbital): 0. Absent; 1. Present.

30. Palpebral/supraorbital: 0. Free, projects into orbit from contact with lacrimal/prefrontal; 1. Incorporated into orbital margin.

31. Palpebral, shape in dorsal view: 0. Rod-shaped; 1. Plate-like with wide base.

32. Palpebral/supraorbital, number: 0. One; 1. Two; 2. Three.

33. Free palpebral, length, relative to anteroposterior width of orbit: 0. Does not traverse entire width of orbit; 1. Traverses entire width of orbit.

34. Exclusion of the jugal from the posteroventral margin of the external antorbital fenestra by lacrimal–maxilla contact: 0. Absent; 1. Present.

35. Anterior ramus of jugal, proportions: 0. Deeper than wide, but not as deep as the posterior ramus of the jugal; 1. Wider than deep; 2. Deeper than the posterior ramus of the jugal.

36. Widening of the skull across the jugals, chord from frontal orbital margin to extremity of jugal is more than minimum interorbital width: 0. Absent; 1. Present, skull has a triangular shape in dorsal view.

37. Position of maximum widening of the skull: 0. Beneath the jugal–postorbital bar; 1. Posteriorly, beneath the infratemporal fenestra.

38. Jugal (or jugal–epijugal) ridge dividing the lateral surface of the jugal into two planes: 0. Absent; 1. Present.

39. Epijugal: 0. Absent; 1. Present.

40. Jugal boss: 0. Absent; 1. Present.

41. Node-like ornamentation on jugal, mostly on, or ventral to, the jugal–postorbital bar: 0. Absent; 1. Present.

42. Jugal–postorbital bar, width broader than infratemporal fenestra: 0. Absent; 1. Present.

43. Jugal–postorbital joint: 0. Elongate scarf joint; 1. Short butt joint.

44. Jugal, form of postorbital process: 0. Not expanded dorsally; 1. Dorsal portion of postorbital process is expanded posteriorly.

45. Jugal–squamosal contact above infratemporal fenestra: 0. Absent; 1. Present.

46. Jugal posterior ramus, forked: 0. Absent; 1. Present.

47. Jugal, posterior ramus: 0. Forms anterior and ventral margin of infratemporal fenestra; 1. Forms part of posterior margin, expands towards squamosal.

48. Jugal–quadratojugal contact: 0. Overlapping; 1. Tongue-and-groove.

49. Postorbital, orbital margin: 0. Relatively smooth curve; 1. Prominent and distinct projection into orbit.

50. Postorbital: 0. T-shaped; 1. Triangular and plate-like.

51. Postorbital–parietal contact: 0. Absent, or very narrow; 1. Broad.

52. Contact between dorsal process of quadratojugal and descending process of the squamosal: 0. Present; 1. Absent.

53. Quadratojugal, shape: 0. L-shaped, with elongate anterior process; 1. Subrectangular with long axis vertical, short, deep anterior process.

54. Quadratojugal, ventral margin: 0. Approaches the mandibular condyle of the quadrate; 1. Well-removed from the mandibular condyle of the quadrate.

55. Quadratojugal, orientation: 0. Faces laterally; 1. Faces posterolaterally.

56. Quadratojugal, transverse width: 0. Mediolaterally flattened; 1. Transversely expanded and triangular in coronal section.

57. Prominent oval fossa on pterygoid ramus of quadrate: 0. Absent; 1. Present.

58. Quadrate lateral ramus: 0. Present; 1. Absent.

59. Quadrate shaft: 0. Anteriorly convex in lateral view; 1. Reduced in anteroposterior width and straight in lateral view.

60. Paraquadratic foramen or notch, size: 0. Absent or small, opens between quadratojugal and quadrate; 1. Large.

61. Paraquadratic foramen, orientation: 0. Posterolateral aspect of quadrate shaft; 1. Lateral aspect of quadrate or quadratojugal.

62. Paraquadratic foramen, position: 0. On quadrate-quadratojugal boundary; 1. Located within quadratojugal.

63. Quadrate mandibular articulation: 0. Quadrate condyles subequal in size; 1. Medial condyle is larger than lateral condyle; 2. Lateral condyle is larger than medial.

64. Paired frontals: 0. Short and broad; 1. Narrow and elongate (more than twice as long as wide).

65. Supratemporal fenestrae: 0. Open; 1. Closed.

66. Supratemporal fenestrae, anteroposteriorly elongated: 0. Absent, fenestrae are subcircular to oval in shape 1. Present.

67. Parietal septum, form: 0. Narrow and smooth; 1. Broad and rugose.

68. Parietosquamosal shelf: 0. Absent; 1. Present.

69. Parietosquamosal shelf, extended posteriorly as distinct frill: 0. Absent; 1. Present.

70. Composition of the posterior margin of the parietosquamosal shelf: 0. Parietal contributes only a small portion to the posterior margin;1. Parietal makes up at least 50 per cent of the posterior margin.

71. Postorbital–squamosal bar: 0. Bar-shaped; 1. Broad, flattened.

72. Postorbital–squamosal tubercle row: 0. Absent; 1. Present.

73. Enlarged tubercle row on the posterior squamosal: 0. Absent; 1. Present.

74. Frontal and parietal dorsoventral thickness: 0. Thin; 1. Thick.

75. Paroccipital processes: 0. Extend laterally and are slightly expanded distally; 1. Distal end pendent and ventrally extending.

76. Paroccipital processes, proportions: 0. Short and deep (height ≥ 1/2 length); 1. Elongate and narrow.

77. Posttemporal foramen/fossa, position: 0. Totally enclosed with the paroccipital process; 1. Forms a notch in the dorsal margin of the paroccipital process, enclosed dorsally by the squamosal.

78. Supraoccipital, contribution to dorsal margin of foramen magnum: 0. Forms entire dorsal margin of foramen magnum; 1. Exoccipital with medial process that restricts the contribution of the supraoccipital.

79. Basioccipital, contribution to the border of the foramen magnum: 0. Present; 1. Absent, excluded by exoccipitals.

80. Basisphenoid: 0. Longer than, or subequal in length to, basioccipital; 1. Shorter than basioccipital.

81. Prootic–basisphenoid plate: 0. Absent; 1. Present.

82. Basal tubera, shape: 0. Knob-shaped; 1. Plate-shaped.

83. Basipterygoid processes, orientation: 0. Anteroventral; 1. Ventral; 2. Posteroventral.

84. Premaxilla–vomeral contact: 0. Present; 1. Absent, excluded by midline contact between maxillae.

85. Dorsoventrally deep (deeper than 50% of snout depth) median palatal keel formed of the vomers, pterygoids and palatines: 0. Absent; 1. Present.

86. Pterygovomerine keel, length: 0. Less than 50% of palate length; 1. More than 50% of palate length.

87. Pterygoid–maxilla contact at posterior end of tooth row: 0. Absent; 1. Present.

88. Pterygoquadrate rami, posterior projection of ventral margin: 0. Weak; 1. Pronounced.

89. Cortical remodeling of surface of skull dermal bone: 0. Absent; 1. Present.

90. Predentary: 0. Absent; 1. Present.

91. Predentary size: 0. Short, posterior premaxillary teeth oppose anterior dentary teeth; 1. Roughly equal in length to the premaxilla, premaxillary teeth only oppose predentary.

92. Predentary, rostral end in dorsal view: 0. Rounded; 1. Pointed.

93. Predentary, oral margin: 0. Relatively smooth; 1. Denticulate.

94. Tip of predentary in lateral view: 0. Does not project above the main body of predentary; 1. Strongly upturned relative to main body of predentary.

95. Predentary, ventral process: 0. Single; 1. Bilobate.

96. Predentary, ventral process: 0. Present, well-developed; 31. Very reduced or absent.

97. Dentary symphysis: 0. V-shaped; 1. Spout shaped.

98. Dentary tooth row (and edentulous anterior portion) in lateral view: 0. Straight; 1. Anterior end downturned.

99. Dorsal and ventral margins of the dentary: 0. Converge anteriorly; 1. Subparallel.

100. Ventral flange on dentary: 0. Absent; 1. Present.

101. Coronoid process: 0. Absent or weak, posterodorsally oblique, depth of mandible at coronoid is less than 140% depth of mandible beneath tooth row; 1. Well-developed, distinctly elevated, depth of mandible at coronoid is more than 180% depth of mandible beneath tooth row.

102. Anterodorsal margin of coronoid process formed by posterodorsal process of dentary: 0. Absent; 1. Present.

103. Coronoid process, position: 0. Posterior to dentition; 1. Lateral to dentition.

104. External mandibular fenestra, situated on dentary-surangular-angular boundary: 0. Present; 1. Absent.

105. Small fenestra positioned dorsally on the surangular-dentary joint: 0. Absent; 1. Present.

106. Ridge or process on lateral surface of surangular, anterior to jaw suture: 0. Absent; 1. Present, anteroposteriorly extended ridge; 2. Present, dorsally directed finger-like process.

107. Retroarticular process: 0. Elongate; 1. Rudimentary or absent.

108. Node-like ornamentation of the dentary and angular: 0. Absent; 1. Present.

109. Level of jaw joint: 0. Level with tooth row, or weakly depressed ventrally; 1. Strongly depressed ventrally, more than 40% of the height of the quadrate is below the level of the maxilla.

110. Mandibular osteoderm: 0. Absent; 1. Present.

111. Premaxillary teeth: 0. Present; 1. Absent, premaxilla edentulous.

112. Premaxillary teeth, number: 0. Six; 1. Five; 2. Four; 3. Three; 4. Two; 5. One.

113. Premaxillary teeth, crown expanded above root: 0. Crown is unexpanded mesiodistally above root, no distinction between root and crown is observable; 1. Crown is at least moderately expanded above root.

114. Premaxillary teeth increase in size posteriorly: 0. Absent, all premaxillary teeth subequal in size; 1. Present, posterior premaxillary teeth are significantly larger in size than anterior teeth.

115. Maxillary and dentary crowns, shape: 0. Apicobasally tall and blade-like; 1. Apicobasally short and sub-triangular; 2. Diamond-shaped.

116. Maxillary/dentary teeth, marginal ornamentations: 0. Fine serrations set at right angles to the margin of the tooth; 1. Coarse serrations (denticles) angle upwards at 45 degrees from the margin of the tooth.

117. Enamel on maxillary/dentary teeth: 0. Symmetrical; 1. Asymmetrical.

118. Apicobasally extending ridges on maxillary/dentary teeth: 0. Absent; 1. Present.

119. Apicobasally extending ridges on lingual/labial surfaces of maxillary/dentary crowns confluent with marginal denticles: 0. Absent; 1. Present.

120. Prominent primary ridge on labial side of maxillary teeth: 0. Absent; 1. Present.

121. Prominent primary ridge on lingual side of dentary teeth: 0. Absent; 1. Present.

122. Position of maxillary/dentary primary ridge: 0. Centre of the crown surface, giving the crown a relatively symmetrical shape in lingual/labial view; 1. Offset, giving crown asymmetrical appearance.

123. At least moderately developed labiolingual expansion of crown (‘cingulum’) on maxillary/dentary teeth: 0. Present; 1. Absent.

124. Heterodont dentary dentition: 0. No substantial heterodonty is present in dentary dentition; 1. Single, enlarged, caniform anterior dentary tooth, crown is not mesiodistally expanded above root; 2. Anterior dentary teeth are strongly recurved and caniform, but have crowns expanded mesiodistally above their roots and are not enlarged relative to other dentary teeth.

125. Peg-like tooth located anteriorly within dentary, lacks denticles, strongly reduced in size: 0. Absent; 1. Present.

126. Alveolar foramina (‘special foramina’) medial to maxillary/dentary tooth rows: 0. Present; 1. Absent.

127. Recurvature in maxillary and dentary teeth: 0. Present; 1. Absent.

128. Overlap of adjacent crowns in maxillary and dentary teeth: 0. Absent; 1. Present.

129. Crown is mesiodistally expanded above root in cheek teeth: 0. Absent; 1. Present.

130. Position of maximum apicobasal crown height in dentary/maxillary tooth rows: 0. Anterior portion of tooth row; 1. Central portion of tooth rows; 2. Caudal portion of tooth rows.

131. Close-packing and quicker replacement eliminates spaces between alveolar border and crowns of adjacent functional teeth: 0. Absent; 1. Present.

132. Fusion between the intercentum of the atlas and the neural arches: 0. Absent; 1. Present.

133. Epipophyses on anterior (postaxial) cervicals: 0. Present; 1. Absent.

134. Cervicals 4-9, form of central surfaces: 0. Amphicoelous; 1. At least slightly opisthocoelous.

135. Cervical number: 0. Seven/eight; 1. Nine; 2. Ten or more.

136. Articulation between the zygapophyses of dorsal vertebrae: 0. Flat; 1. Tongue-and-groove.

137. Dorsals, number: 0. 12–13; 1. 15; 2. 16 or more.

138. Sacrals, number: 0. Two; 1. Three; 2. Four/five; 3. Six or more.

139. Sacrum, accessory articulation with pubis: 0. Absent; 1. Present.

140. Posterior sacral ribs are considerably longer than anterior sacral ribs: 0. Absent;

1. Present.

141. Anterior caudal vertebrae, length of transverse processes relative to neural spine height: 0. Subequal; 1. Longer than neural spine.

142. Proximal caudal neural spines: 0. Height the same or up to 50 per cent taller than the centrum; 1. More than 50 per cent taller than the centrum.

143. Elongate tail (59 or more caudals): 0. Absent; 1. Present.

144. Chevron shape: 0. Rod-shaped, often with slight distal expansion; 1. Strongly asymmetrically expanded distally, width greater than length in mid caudals.

145. Sternal segments of the anterior dorsal ribs: 0. Unossified; 1. Ossified.

146. Gastralia: 0. Present; 1. Absent.

147. Ossified clavicles: 0. Absent; 1. Present.

148. Sternal plates, shape: 0. Absent; 1. Kidney-shaped; 2. Shafted or hatchet-shaped (rod-like posterolateral process, expanded anterior end).

149. Proportions of humerus and scapula: 0. Scapula longer or subequal to the humerus; 1. Humerus substantially longer than the scapula.

150. Scapula blade, length relative to minimum width: 0. Relatively short and broad, length is 5-8 times minimum width; 1. Elongate and strap-like, length is at least 9 times the minimum width.

151. Scapula acromion shape: 0. Weakly developed or absent; 1. Well-developed spine-like.

152. Scapula, blade-shape: 0. Strongly expanded distally; 1. Weakly expanded, near parallel-sided.

153. Humeral length: 0. More than 60% of femoral length; 1. Less than 60% of femoral length.

154. Deltopectoral crest development: 0. Well-developed, projects anteriorly as a distinct flange; 1. Rudimentary, is at most a thickening on the anterolateral margin of the humerus.

155. Humeral shaft form, in anterior or posterior view: 0. Relatively straight; 1. Strongly bowed laterally along length.

156. Longest manual phalanx as percentage of length of humerus: 0. Less than 10% ; 1. More than 15%.

157. Metacarpals with block-like proximal ends: 0. Absent; 1. Present.

158. Metacarpals 1 and 5: 0. Substantially shorter in length than metacarpal 3; 1. Subequal in length to metacarpal 3.

159. Penultimate phalanx of the second and third fingers: 0. Shorter than first phalanx; 1. Longer than the first phalanx.

160. Manual digit 3, number of phalanges: 0. Four; 1. Three or fewer.

161. Manual digits 2–4: 0. First phalanx relatively short compared to second phalanx; 1. First phalanx more than twice the length of the second phalanx.

162. Extensor pits on the dorsal surface of the distal end of metacarpals and manual phalanges: 0. Absent or poorly developed; 1. Deep, well-developed.

163. Manual unguals strongly recurved with prominent flexor tubercle: 0. Absent; 1. Present.

164. Acetabulum: 0. At least a small perforation; 1. Completely closed.

165. Preacetabular process, shape / length: 0. Short, tab-shaped, distal end is posterior to pubic peduncle; 1. Elongate, strap-shaped, distal end is anterior to pubic peduncle.

166. Preacetabular process, length: 0. Less than 50% of the length of the ilium; 1. More than 50% of the length of the ilium.

167. Preacetabular process, lateral deflection: 0. 10–20 degrees from midline; 1. More than 30 degrees.

168. Dorsal margin of preacetabular process and dorsal margin of ilium above acetabulum: 0. Narrow, not transversely expanded; 1. Dorsal margin is transversely expanded to form a narrow shelf.

169. In dorsal view preacetabular process of the ilium expands mediolaterally towards its distal end: 0. Absent; 1. Present.

170. Dorsal margin of the ilium in lateral view: 0. Relatively straight or slightly convex; 1. Sinuous, postacetabular process is strongly upturned.

171. Subtriangular process extending medially from the dorsal margin of the iliac blade: 0. Absent; 1. Present.

172. Subtriangular process, form and position: 0. Short and tab-like, above acetabulum; 1. Elongate and flange-like, on postacetabular process.

173. Brevis shelf & fossa: 0. Fossa faces ventrolaterally and shelf is near vertical and visible in lateral view along entire length, creating a deep postacetabular portion; 1. Fossa faces ventrally and posterior of shelf portion cannot be seen in lateral view.

174. Length of the postacetabular process as a percentage of the total length of the ilium: 0. 20% or less; 1. 25-35%; 2. More than 35%.

175. Medioventral acetabular flange of ilium, partially closes the acetabulum: 0. Present; 1. Absent.

176. Supra-acetabular ‘crest’ or ‘flange’: 0. Present; 1. Absent.

177. Ischial peduncle of the ilium: 0. Projects ventrally; 1. Broadly swollen, projects ventrolaterally.

178. Pubic peduncle of ilium: 0. Large, elongate, robust; 1. Reduced in size, shorter in length than ischial peduncle.

179. Pubic peduncle of ischium, shape: 0. Transversely compressed; 1. Dorsoventrally compressed.

180. Ischium, shape of shaft: 0. Relatively straight; 1. Gently curved along length; 2. Distinct bend at midlength.

181. Ischial shaft, cross-section: 0. Compressed mediolaterally; 1. Subcircular and bar-like.

182. Ischial shaft: 0. Expands weakly, or is parallel-sided, distally; 1. Distally expanded into a distinct ‘foot’; 2. Tapers distally.

183. Groove on the dorsal margin of the ischium: 0. Absent; 1. Present.

184. Tab-shaped obturator process on ischium: 0. Absent; 1. Present.

185. Ischial symphysis, length: 0. Ischium forms a median symphysis with the opposing blade along at least 50% of its length; 1. Ischial symphysis present distally only.

186. Pubis, orientation: 0. Anteroventral; 1. Rotated posteroventrally to lie alongside the ischium (opisthopubic).

187. Shaft of pubis (postpubis), shape in cross-section: 0. Blade-shaped; 1. Rod-shaped.

188. Shaft of pubis (postpubis), length: 0. Approximately equal in length to the ischium; 1. Reduced, extends for half or less the length of the ischium.

189. Reduction of postpubic shaft: 0. Postpubic shaft extends for around half the length of ischium; 1. Postpubic shaft is very short or absent.

190. Body of pubis, size: 0. Relatively large, makes substantial contribution to the margin of the acetabulum; 1. Reduced in size, rudimentary, nearly excluded from the acetabulum.

191. Body of the pubis, massive and dorsolaterally rotated so that obturator foramen is obscured in lateral view: 0. Absent; 1. Present.

192. Prepubic process: 0. Absent; 1. Present.

193. Prepubic process: 0. Compressed mediolaterally, dorsoventral height exceeds mediolateral width; 1. Rod-like, mediolateral width exceeds dorsoventral height; 2. Dorsoventrally compressed; 3. Twisted along length – dorsoventrally compressed at its base and transversely compressed distally.

194. Prepubic process, length: 0. Stub-like and poorly developed, extends only a short distance anterior to the pubic peduncle of the ilium; 1. Elongated into distinct anterior process.

195. Prepubic process, extends beyond distal end of preacetabular process of ilium: 0. Absent; 1. Present.

196. Extent of pubic symphysis: 0. Elongate; 1. Restricted to distal end of pubic blade, or absent.

197. Femoral shape in medial/lateral view: 0. Bowed anteriorly along length; 1. Straight.

198. Femoral head: 0. Confluent with greater trochanter, fossa trochanteris is groove-like; 1. Fossa trochanteris is modified into distinct constriction separating head and greater trochanter.

199. ‘Anterior’ or ‘lesser’ trochanter, morphology: 0. Absent; 1. Trochanteric shelf ending in a small, pointed, spike; 2. Broadened, prominent, ‘wing’ or ‘blade’ shaped, sub-equal in anteroposterior width to greater trochanter; 3. Reduced anteroposterior width, closely appressed to the expanded greater trochanter.

200. Level of most proximal point of anterior trochanter relative to level of proximal femoral head: 0. Anterior trochanter is positioned distally on the shaft, and separated from ‘dorsolateral’ trochanter/greater trochanter by deep notch visible in medial view; 1. Anterior trochanter positioned proximally, approaches level of proximal surface of femoral head, closely appressed to ‘dorsolateral’/greater trochanter (no notch visible in medial view).

201. Fourth trochanter of femur, shape: 0. Low eminence, or absent; 1. Prominent ridge; 2. Pendent.

202. Fourth trochanter, position: 0. Located entirely on proximal half of femur; 1. Positioned at midlength, or distal to midlength.

203. Anterior (extensor) intercondylar groove on distal end of femur: 0. Absent; 1. Present.

204. Posterior (flexor) intercondylar groove of the femur: 0. Fully open; 1. Medial condyle inflated laterally, partially covers opening of flexor groove.

205. Lateral condyle of distal femur, position and size in ventral view: 0. Positioned relatively laterally, and slightly narrower in width than the medial condyle; 1. Strongly inset medially, reduced in width relative to medial condyle.

206. Distal tibia: 0. Subquadrate, posterolateral process is not substantially developed; 1. Elongate posterolateral processs, backs fibula.

207. Fibular facet on the lateral margin of the proximal surface of the astragalus: 0. Large; 1. Reduced to small articulation.

208. Calcaneum, proximal surface: 0. Facet for tibia absent; 1. Well-developed facet for tibia present.

209. Medial distal tarsal: 0. Articulates distally with metatarsal 3 only; 1. Articulates distally with metatarsals 2 and 3.

210. Metatarsal arrangement: 0. Compact, closely appressed to one another along 50-70% of their length, spread distally; 1. Contact each other only at proximal ends, spread strongly outwards distally.

211. Digit 1: 0. Metatarsal 1 robust and well-developed, distal end of phalanx 1-1 projects beyond the distal end of metatarsal 2; 1. Metatarsal 1 reduced & proximally splint like, end of phalanx 1-1 does not extend beyond the end of metatarsal 2; 2. Metatarsal 1 reduced to a vestigal splint or absent, does not bear digits.

212. Pedal digit 4 phalangeal number: 0. Five; 1. Four or fewer.

213. Metatarsal 5, length: 0. More than 50 per cent of metatarsal 3; 1. Less than 25 per cent of metatarsal 3.

214. Metatarsal 5: 0. Bears digits; 1. Lacks digits.

215. Pedal unguals, shape: 0. Tapering, narrow pointed, claw-like; 1. Wide, blunt, hoof-like.

216. Epaxial ossified tendons present along vertebral column: 0. Absent; 1. Present.

217. Ossified hypaxial tendons, present on caudal vertebrae: 0. Absent; 1. Present.

218. Ossified tendons, arrangement: 0. Longitudinally arranged; 1. Basket-like arrangement of fusiform tendons in caudal region; 2. Double-layered lattice.

219. Parasagittal row of dermal osteoderms on the dorsum of the body: 0. Absent; 1. Present.

220. Lateral row of keeled dermal osteoderms on the dorsum of the body: 0. Absent; 1. Present.

221. U-shaped cervical / pectoral collars composed of contiguous keeled osteoderms: 0. Absent; 1. Present.

222. Wear facets on teeth: 0. Absent or sporadically developed; 1. Systematic development of wear facets along the entire tooth row.

223. Head of humerus is separated from prominent medial tubercle on proximal surface by a groove: 0. Absent; 1. Present.

224. Pendent fourth trochanter, rod-like with subparallel anterior and posterior surfaces: 0. Absent; 1. Present.

225. Fibula, distal end is strongly reduced and splint-like: 0. Absent; 1. Present.

226. Astragalus and calcaneum are indistinguishably fused to one another: 0. Absent; 1. Present.

227. Maximum expansion of distal tibia relative to proximal: 0. Distal tibia is considerably less expanded than proximal; 1. Maximum expansion of distal tibia is subequal to that of proximal tibia.

**New characters**

C. 228. Number of dentary teeth: 10 or fewer (0); 11–13 (1); 14–17 (2); 18 or more (3) (modified from Weishampel *et al*. 2003: ch. 30; ordered)

C. 229. Number of ridges on dentary crowns: fewer than 10 (0) more than 10, and often more than 17 (1) (Weishampel *et al*. 2003: ch. 32)

C. 230. Dentary, posterolateral surface, depression: absent (0); present (1) (new character)

C. 231. Development of the sternal process of the coracoid: short and broad (0); extremely elongated and narrow (ratio greater than 0.80) (1) (Weishampel *et al*. 2003: ch. 44).

C. 232. Proximal end of the humerus in anterior or posterior view, lateral border between head and deltopectoral crest: straight or gently convex (0); concave (1) (new character)

C. 233. Ulna, distal end: directed ventrally in medial or lateral view (0); curves gently posteriorly in medial or lateral view (1) (new character)
